# Predictive Performance of Inflammatory Markers for Severe Disease Progression in Measles: A Retrospective Cohort Analysis

**DOI:** 10.3390/microorganisms14102314

**Published:** 2026-10-10

**Authors:** Alexandra Herlo, Adelina-Raluca Marinescu, Ruxandra Laza, Ioana-Melinda Luput-Andrica, Voichita Elena Lazureanu, Lucian-Flavius Herlo, Aneta-Rada Dobrin, Kersztin Lorette Csiki, Daniela-Ica Rosoha, Claudia Raluca Balasa Virzob, Daniela Teodora Marti, Talida Georgiana Cut

**Affiliations:** 1 Department XIII, Discipline of Infectious Diseases, “Victor Babes” University of Medicine and Pharmacy Timisoara, 2 Eftimie Murgu Square, 300041 Timisoara, Romania; alexandra.mocanu@umft.ro (A.H.); adelina.marinescu@umft.ro (A.-R.M.); laza.ruxandra@umft.ro (R.L.); talida.cut@umft.ro (T.G.C.); 2 Doctoral School, “Victor Babes” University of Medicine and Pharmacy Timisoara, 300041 Timisoara, Romania; flavius.herlo@umft.ro (L.-F.H.); aneta.goia@umft.ro (A.-R.D.); kersztin.csiki@umft.ro (K.L.C.); daniela.rosoha@umft.ro (D.-I.R.); 3 Department of Clinic Nursing, “Victor Babes” University of Medicine and Pharmacy Timisoara, E. Murgu Square, Nr. 2, 300041 Timisoara, Romania; virzob.claudia@umft.ro; 4 Department of Biology and Life Sciences, Faculty of Medicine, Vasile Goldis Western University of Arad, 310025 Arad, Romania; marti.teodora@uvvg.ro

**Keywords:** measles, interleukin-6, ferritin, vaccination, biomarkers, ROC analysis, severity, risk stratification, length of stay, Cox regression, pediatric pulmonology, acute infection, vaccine-preventable disease

## Abstract

The recent European measles resurgence has driven record-high hospitalization, yet objective tools for early risk stratification remain scarce. This study evaluated the diagnostic accuracy of routine admission inflammatory biomarkers for predicting severe disease and prolonged hospitalization. We retrospectively analyzed 509 patients with laboratory-confirmed measles hospitalized during a Romanian outbreak (November 2023–August 2025). Severe disease was defined via a clinical composite endpoint (intensive-care admission, mechanical ventilation, supplemental oxygen, acute respiratory failure, or sepsis). Biomarker discriminative performance was assessed using receiver-operating-characteristic (ROC) curves, Youden-optimal cut-offs, and DeLong tests. Multivariable logistic and Cox proportional-hazards regression evaluated independent predictors of severity and time to discharge. The cohort exhibited a pediatric predominance (median age: 3.0 years; 20% infants) with an 82% unvaccinated rate; 33% developed severe disease. Interleukin-6 (IL-6) demonstrated superior diagnostic accuracy (AUC 0.863, 95% CI 0.820–0.898; cut-off ≥ 7 pg/mL, sensitivity 73%, specificity 93%), significantly outperforming ferritin (AUC 0.800) and conventional markers (all *p* < 0.001). Multivariable modeling established IL-6 and ferritin as the sole independent predictors of severity. All intensive-care admissions occurred exclusively for unvaccinated patients (5.0% vs. 0%). Severe disease, pneumonia, male sex, unvaccinated status and elevated C-reactive protein (CRP) independently predicted prolonged hospitalization. Admission IL-6 and ferritin serve as robust, independent prognostic biomarkers that accurately identify high-risk measles patients, providing objective criteria with which to optimize clinical triage and inpatient management during outbreaks.

## 1. Introduction

The resurgence of measles has emerged as a critical public health challenge within the WHO European Region, recording 127,350 cases in 2024—the highest since 1997 and a twofold increase from 2023, with children under five accounting for over 40% of the burden [1]. Despite the availability of safe and effective vaccines, global immunization gaps, pandemic-related service disruptions, and rising vaccine hesitancy continue to drive dangerous outbreaks [2,3]. Romania has become the epicenter of this epidemic, exhibiting the highest national caseload, particularly among infants under one year of age who are ineligible for routine primary immunization [1,4]. In response to the severity of this national outbreak, the Romanian Ministry of Health implemented an emergency public health measure, temporarily lowering the age of administration for the first dose of the measles–mumps–rubella (MMR) vaccine from the standard 12 months to 9 months (commonly referred to as “dose zero”).

Characterized by high contagiousness (*R*_0_ of 12–18 [5,6]), this “old-new” disease challenges healthcare systems worldwide, with approximately 86% of documented European cases remaining unvaccinated [7,8]. Although case counts declined throughout 2025, the re-establishment of epidemic transmission in multiple nations confirms that measles remains a persistent and urgent public health threat [9]. Moreover, these outbreaks represent a significant paradigm shift; despite decades of progress toward elimination, the combination of pandemic-related disruptions, vaccine hesitancy, and waning clinical awareness has facilitated the global re-emergence of this pathogen [3]. Recent global analyses designate this resurgence as an urgent international health security threat that exposes critical vulnerabilities in public health surveillance [10,11]. Managing measles is complicated by the absence of specific antiviral therapy [12], making early prognostic identification essential given the frequency of severe pulmonary and neurological complications [13].

**Figure 1:**
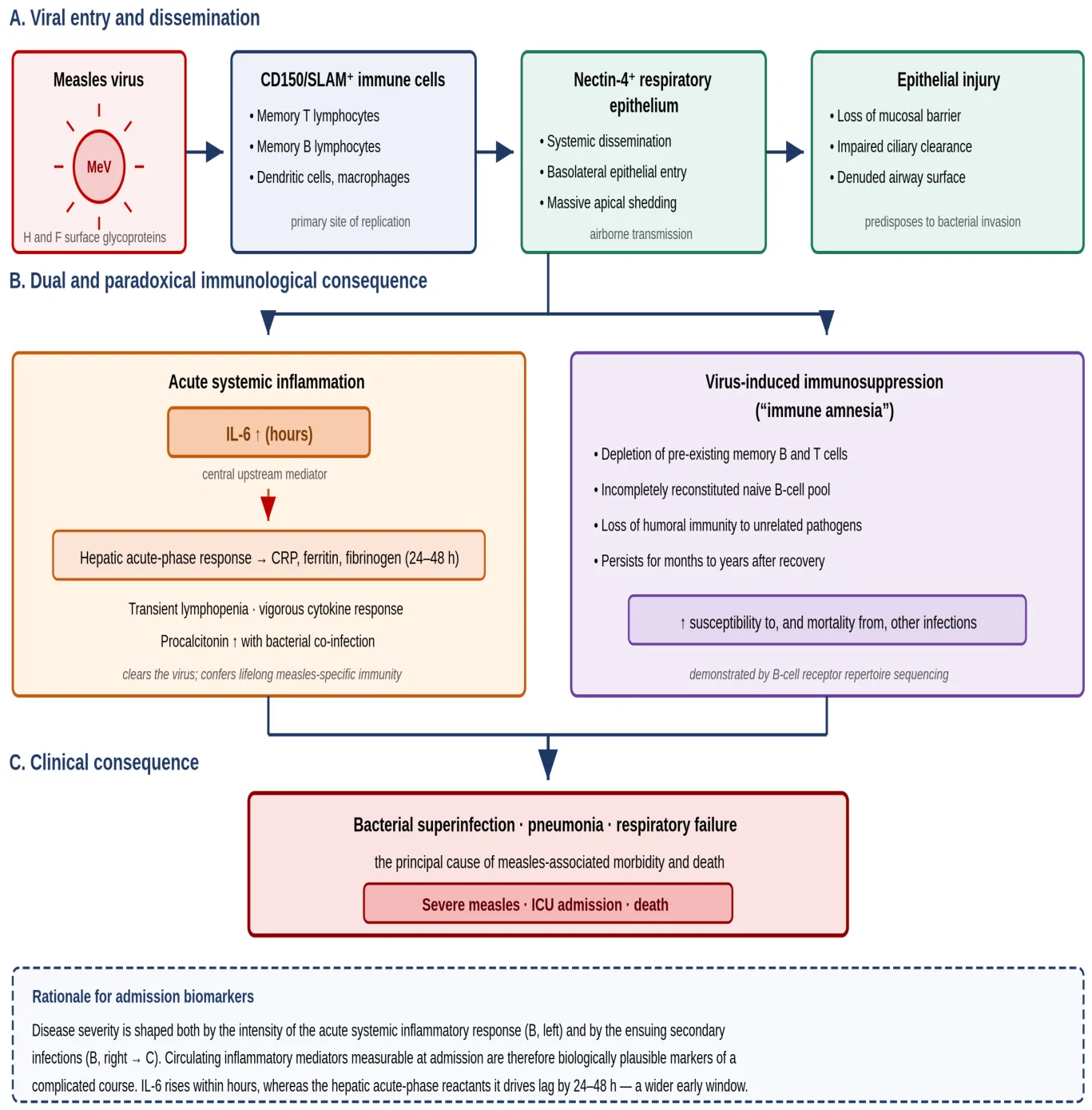
Schematic overview of measles-virus-induced immunopathogenesis, dual immunological consequences, and clinical endpoints, establishing the biological rationale for admission inflammatory biomarkers.

Measles remains a highly transmissible yet vaccine-preventable disease, where persistent outbreaks underscore the urgent need for high immunization coverage and herd immunity to mitigate severe complications [14]. In the absence of specific antiviral therapies, clinical management is strictly supportive, focusing on fever control, hydration, and the treatment of secondary complications, most notably pneumonia, otitis media, laryngotracheobronchitis, and gastroenteritis; mortality risk is disproportionately higher among vulnerable populations, including young children, pregnant women and the immunocompromised [15,16,17,18]. While recent national epidemiological evaluations have characterized baseline clinical and laboratory manifestations and identified procalcitonin as a predictor of pneumonia [19,20,21,22], the rapid clinical stratification of patients, specifically distinguishing those at risk of severe deterioration, remains a persistent challenge. Validated, objective tools for early prognostic risk assessment are currently limited, making the refinement of early identification strategies and heightened clinical vigilance essential [23] (see Figure 1).

The clinical pathophysiology of measles features a paradoxical immunological profile, where viral entry targets CD150/SLAM-expressing immune cells before secondary epithelial dissemination via nectin-4 [15,24]. While the subsequent robust systemic inflammatory response and cytokine upregulation facilitate viral clearance and lifelong immunity [24,25], the virus simultaneously depletes pre-existing memory lymphocytes and impairs naïve B-cell repertoire reconstitution [26,27]. This phenomenon, known as “immune amnesia,” combined with respiratory epithelial injury, induces prolonged susceptibility to secondary infections [6,18,24,26,28]. Because measles-associated morbidity and mortality stem from this acute inflammation and vulnerability to bacterial superinfection, circulating inflammatory mediators serve as biologically robust markers of clinical complexity.

Systemic inflammatory biomarkers are valuable prognostic tools due to their cost-effectiveness and rapid clinical availability. In severe viral syndromes such as COVID-19, IL-6 and CRP are established predictors of critical care requirements, with IL-6 often demonstrating superior discriminatory power compared to conventional hematological markers [29,30]. IL-6 further provides prognostic utility in pediatric community-acquired pneumonia [31], while ferritin and procalcitonin serve as established outcome indicators across various severe viral infections [32]. As a central upstream orchestrator of the systemic inflammatory cascade [33], IL-6 represents a prime candidate for prognostic stratification in measles. However, the diagnostic accuracy and clinical thresholds of these biomarkers for measles severity remain undefined. To address this knowledge gap, we analyzed a large, single-center cohort from the Romanian outbreak to (i) quantify the diagnostic accuracy of routine admission biomarkers for severe measles and derive clinically applicable thresholds; (ii) identify independent predictors of a severe clinical course; and (iii) determine the factors associated with prolonged hospitalization.

## 2. Materials and Methods

### 2.1. Methodological Framework and Ethical Approval

We conducted a single-center, retrospective observational cohort study with a nested prognostic-accuracy component at the “Dr. Victor Babes” Clinical Hospital of Infectious Diseases and Pneumophthisiology, Timisoara, a tertiary referral center serving western Romania. The study period, extending from November 2023 to August 2025, encompassed the peak of the national measles epidemic wave. This report adheres to the STARD 2015 guidelines for diagnostic accuracy studies, the TRIPOD statement for multivariable prediction models, and the STROBE statement for observational research.

The protocol was conducted in accordance with the Declaration of Helsinki and the EU General Data Protection Regulation. Approval was granted by the Ethics Committee of the “Dr. Victor Babes” Clinical Hospital of Infectious Diseases and Pneumophthisiology, Timisoara (approval number 98/2023, dated 6 March 2023). Given the retrospective observational design and the exclusive use of pseudonymized medical records, the requirement for individual written informed consent was waived by the Ethics Committee in accordance with national legislation and institutional regulations [34,35]. All medical records were pseudonymized at the time of data extraction and maintained on a secure, encrypted institutional server accessible exclusively to the authorized research personnel.

### 2.2. Eligibility Criteria and Patient Enrolment

All consecutive adult and pediatric patients hospitalized with laboratory-confirmed measles during the study period were eligible for inclusion. Measles infection was confirmed via positive serum anti-measles IgM assay and/or the detection of measles virus RNA using a multiplex respiratory reverse-transcription polymerase chain reaction (RT-PCR) panel (Euroimmun, Lübeck, Germany), adhering strictly to the WHO laboratory case definition [36].

**Figure 2:**
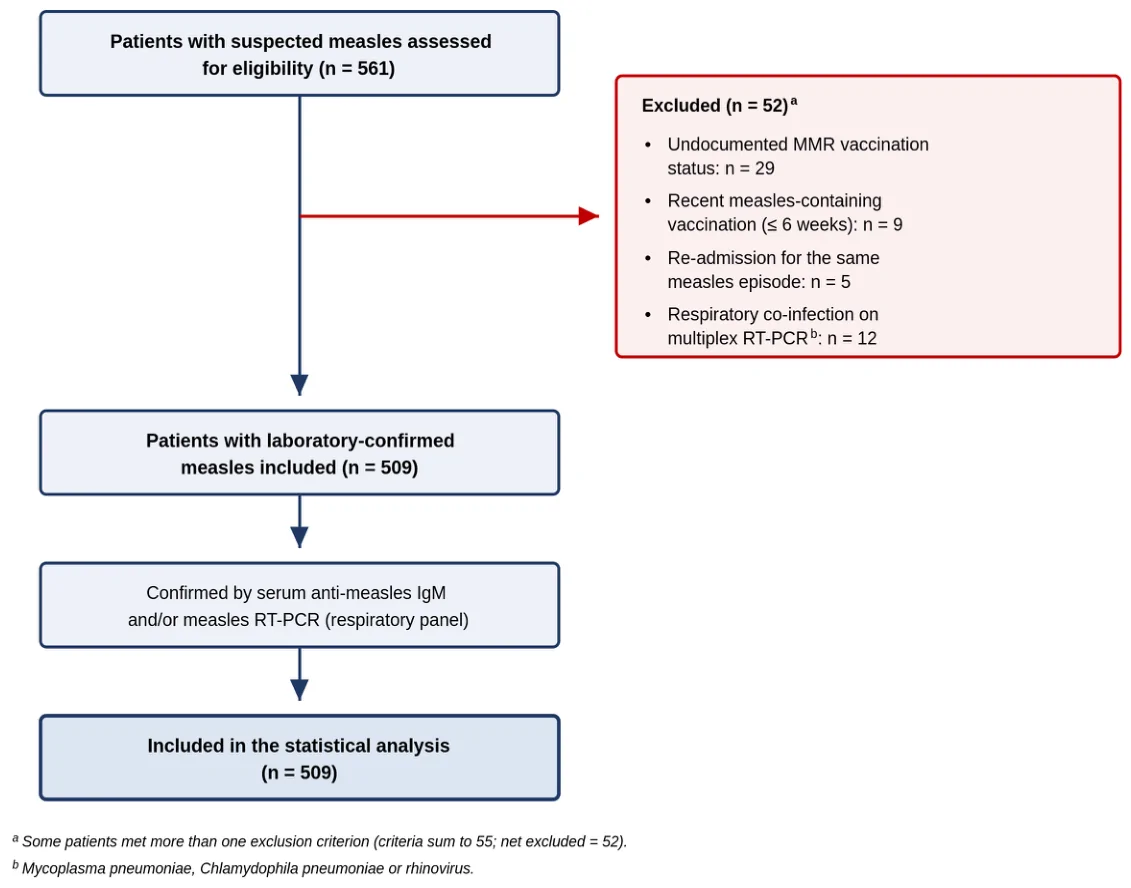
Of 561 assessed patients, 52 were excluded (^**a**^ some met >1 criterion, net *n* = 52): undocumented MMR status (*n* = 29), recent vaccination (*n* = 9), re-admission (*n* = 5), and respiratory co-infection (*n* = 12; ^b^ including *Mycoplasma pneumoniae*, *Chlamydophila pneumoniae*, or *rhinovirus*). A final cohort of 509 patients was analyzed.

Exclusion criteria were applied as follows: (i) receipt of a measles-containing vaccine within six weeks prior to symptom onset to mitigate the risk of misclassifying vaccine-included IgM reactivity; (ii) records corresponding to readmission for the same episode to maintain the independence of clinical observations; (iii) identification of a relevant respiratory co-infection (e.g., *Mycoplasma pneumoniae*, *Chlamydophila pneumoniae*, or rhinovirus) via multiplex panel, as these pathogens serve as significant confounders for admission inflammatory marker levels; or (iv) undocumented MMR vaccination status, given that immunization status was a pre-specified covariate in the multivariable modeling. Of the 561 patients initially screened, 52 met one or more exclusion criteria, resulting in a final analytical cohort of 509 laboratory-confirmed patients (Figure 2).

### 2.3. Data Extraction and Laboratory Assays

Demographic characteristics, vaccination status (MMR), 22 admission laboratory parameters, complications, supportive interventions and clinical outcomes were extracted from electronic medical records into a pre-specified, standardized database. Variable definitions, permissible value ranges, and standard units were explicitly defined in an institutional codebook, with infant age recorded in months subsequently converted to fractional years. All index laboratory parameters, including quantitative IL-6 measurements, were systematically ordered for all consecutive hospitalized patients upon admission as part of the routine institutional protocol implemented during the epidemic wave, thereby eliminating verification bias. IL-6 levels were measured via electrochemiluminescence immunoassay (Elecsys IL-6) using a Cobas e411 analyzer (Roche Diagnostics, Mannheim, Germany), which has a manufacturer-defined reference interval of 0–7 pg/mL. Values falling below the assay’s lower limit of detection (≈1.5 pg/mL) were handled by assigning the respective threshold limit or recorded as baseline low values according to standard laboratory reporting protocols, with decimal values subsequently standardized to one decimal place during database extraction to maintain uniform precision across non-parametric distribution summaries. Procalcitonin was assessed on the same analytical platform via electrochemiluminescence immunoassay (Elecsys BRAHMS PCT, Roche Diagnostics, Mannheim, Germany). CRP and ferritin concentrations were quantified using latex-enhanced immunoturbidimetric assay on an Architect c4000 clinical chemistry analyzer (Abbott Laboratories, Abbott Park, IL, USA), whereas complete blood counts were performed on a Sysmex XN-550 automated hematology analyzer (Sysmex Corporation, Kobe, Japan). Initial clinical evaluations and severity triage were executed by attending physicians upon admission, prior to the availability of biomarker results. The target condition of severe disease (Section 2.4) was subsequently ascertained through objectively documented clinical events within medical charts, independent of the treating physicians’ subjective global assessments. A minor proportion of physiologically implausible laboratory values (18 total data points across specific secondary parameters) were handled as missing data; no data imputation was utilized, and all biomarker analyses were conducted on a complete case, with exact observation counts reported for each comparative evaluation. Specifically, regarding the biomarkers entering the primary ROC and multivariate analyses, IL-6, ferritin, D-dimers, procalcitonin, CRP, leukocytes, and ESR had complete data with zero missing values (*n* = 509), whereas fibrinogen had 2 missing values (*n* = 507).

### 2.4. Clinical Definitions and Outcome Measures

#### 2.4.1. Case Classification and Severity Stratification

Clinical presentations were categorized in accordance with established diagnostic frameworks for measles. Typical (classic) measles was characterized by a prodromal febrile phase accompanied by the classical triad of cough, coryza, and conjunctivitis, followed by the appearance of pathognomonic Koplik spots and a characteristic maculopapular exanthem exhibiting a confluent, cephalocaudal progression [6,16,17,18]. Modified measles was defined as an attenuated clinical phenotype manifesting with a sparse, transient rash and mild systemic symptoms, typically observed in individuals with partial immunological protection, such as infants retaining residual maternal transplacental antibodies or patients who received post-exposure immunoprophylaxis [16,18]. Atypical measles, a historical entity classically associated with recipients of inactivated measles vaccines administered during 1960s, delineated by high fever, a peripherally accentuated rash, and prominent pneumonitis, was not anticipated within this contemporary cohort and is mentioned solely for academic completeness [17,18]. All evaluated cases were laboratory-confirmed and aligned clinically with either typical or modified measles phenotypes.

#### 2.4.2. Primary Outcome and Severity Definition

Severe disease, established as the primary outcome and target condition for the prognostic-accuracy evaluation, was defined a priori strictly on clinical and organ-support criteria, entirely independent of any laboratory biomarker. Because index tests were quantified at admission while the target condition was ascertained over the subsequent clinical course, the study design is inherently prognostic rather than diagnostic, thereby avoiding incorporation bias by construction. A case was classified as severe if it met at least one of the following criteria at any point during hospitalization: (i) admission to the intensive-care unit (ICU); (ii) requirement for endotracheal intubation or invasive mechanical ventilation; (iii) need for supplemental oxygen therapy; (iv) acute respiratory failure; or (v) sepsis.

To ensure diagnostic precision and address potential over-ascertainment, individual components of the composite endpoint were explicitly operationalized using standardized, objective physiological and clinical thresholds:

⇒ Acute respiratory failure was strictly defined by documentation of arterial oxygen saturation (SpO_2_) persistently below 92% in room air, a ratio of arterial oxygen partial to fractional inspired oxygen (PaO_2_/FiO_2_) ≤ 300 mmHg, or documented requirement for non-invasive respiratory support/mechanical ventilation due to acute gasometric decompensation. Furthermore, the requirement for supplemental oxygen therapy was operationalized strictly as the continuous administration of oxygen for a minimum of 24 h to maintain SpO_2_ ≥ 92%, explicitly excluding transient peri-procedural oxygen administration or brief interventions, thereby preventing the over-ascertainment of the severe-disease rate.
⇒ Sepsis was rigorously defined using age-appropriate consensus criteria to ensure diagnostic consistency. For adult patients, sepsis was classified according to the Sepsis-3 criteria, requiring suspected or documented infection accompanied by an acute increase in the Sequential Organ Failure Assessment (SOFA) score of ≥2 points. For the predominantly pediatric cohort, sepsis was defined following the latest international consensus criteria for pediatric sepsis (e.g., the Phoenix Pediatric Sepsis criteria or the International Consensus Conference on Pediatric Sepsis definitions), requiring the presence of systemic inflammatory response syndrome (SIRS) in the setting of suspected infection, accompanied by cardiovascular dysfunction, acute respiratory distress syndrome, or the dysfunction of two or more other organ systems.

Admission to the ICU adhered to pre-specified clinical indications applied by the attending medical team, including acute respiratory failure, oxygen requirements exceeding ward capacity, high-flow nasal cannula or mechanical ventilation requirements, hemodynamic instability, shock or sepsis requiring vasoactive support or invasive monitoring, severe neurological manifestations (e.g., encephalitis, status epilepticus, or depressed consciousness), multi-organ dysfunction syndrome, or any clinical state necessitating continuous monitoring and organ support [16,17,18].

Crucially, admission concentrations of IL-6, procalcitonin, ferritin, CRP and all the other evaluated laboratory parameters were not utilized, either individually or in combination, to define severe disease. Although one component of the composite outcome, ICU admission, represented a clinical decision made by physicians with access to both clinical status and admission laboratory results (rendering it partially dependent on the index tests), the remaining four components consisted of objective physiological events free from this influence. To quantify the potential impact of this partial dependence, a sensitivity analysis excluding ICU admission from the composite outcome was pre-specified (Section 2.5). Furthermore, to address potential heterogeneity within the composite endpoint, a restricted secondary sensitivity analysis focusing strictly on hard clinical endpoints (comprising ICU admission, endotracheal intubation, or gasometrically documented acute respiratory failure) was performed to corroborate the robustness of the primary prognostic findings.

#### 2.4.3. Secondary Outcomes and Clinical Grading

The principal secondary outcome was time to discharge, defined as the temporal interval from hospital admission to discharge. All enrolled patients were discharged alive, resulting in zero mortality censoring. For descriptive subgroup comparisons, disease severity was further categorized into three mutually exclusive clinical phenotypes, with pathological complications strictly restricted to secondary organ-system involvements requiring targeted medical intervention rather than expected cardinal signs or isolated, transient laboratory shifts (thereby excluding routine conjunctivitis and self-limiting transaminase elevations from the complication count):

- Mild (uncomplicated) form: comprising patients presenting with no documented complications and no criteria defining a severe clinical course.
- Moderate (complicated) form: comprising patients exhibiting at least one measles-associated complication (e.g., pneumonia, otitis media, laryngotracheobronchitis, bronchiolitis, hepatic cytolysis, or acute dehydration) in the absence of any severe-course criteria.
- Severe form: comprising patients fulfilling at least one severe-disease criterion as outlined in Section 2.4.2.

This ordinal stratification remains entirely consistent with, and nested within, the binary severe-disease outcome.

#### 2.4.4. Evaluated Exposures and Predictors

Vaccination status was stratified into zero, one, or two documented MMR doses, ascertained via physical immunization booklets and cross-verified against the national electronic immunization registry. Patients whose vaccination status could not be verified through either source were excluded from the cohort (Section 2.2).

### 2.5. Statistical Analysis

Data processing and statistical analysis were performed entirely using MedCalc Statistical Software version 22.026 (MedCalc Software Ltd., Ostend, Belgium; 2025), a tool widely recognized in biomedical research for the accuracy of its ROC curve analysis and regression models.

Data distribution was preliminarily assessed; as continuous variables were predominantly non-normally distributed, they are summarized as medians and interquartile ranges (IQR). Group comparisons were performed using the non-parametric Mann-Whitney U test. Categorical variables are reported as absolute and relative frequencies (percentages), with statistical differences evaluated using Chi-square (X^2^) test or Fisher’s exact test, as appropriate based on standard applicability conditions.

The predictive accuracy of admission biomarkers for subsequent severe disease was assessed through ROC curve analysis, natively implemented within the MedCalc environment. AUC and 95% confidence intervals (CI) were estimated using DeLong method [37]. Optimal decision thresholds (cut-offs) were mathematically determined by calculating the Youden index (*J* = sensitivity + specificity − 1) [38]. Although an a priori sample size calculation was not performed due to the retrospective design, the precision of our primary diagnostic estimates was assessed by examining the confidence intervals; specifically, the 95% confidence interval for the primary AUC of IL-6 (0.863, 95% CI: 0.820–0.898) exhibits a narrow half-width of approximately 0.039, confirming a high degree of statistical precision in our discriminative performance metrics given the final cohort of 509 patients. For these optimal thresholds, the software automatically computed the corresponding sensitivity, specificity, predictive values, and likelihood ratios. Discriminative performance was interpreted according to standardized ROC criteria [39]. Pairwise comparisons between competing biomarker ROC curves were conducted by applying the DeLong test for correlated curves, available in the software’s analysis package [37]. Time to discharge was analyzed using the Kaplan–Meier method, and the differences between survival curves were tested via the log-rank test [40]. Additionally, the Cox proportional-hazards regression model [41] was applied, with results expressed as hazard ratios (HR) for discharge (therefore, an HR < 1 indicates prolonged hospitalization). Because all enrolled patients were discharged alive, resulting in zero mortality censoring, the Cox model functions in this context as a rank-based semiparametric model for length of stay. While alternative analytical frameworks, such as an accelerated failure time (AFT) models, quantile regression, or negative binomial regression, could alternatively model length-to-stay distribution, the Cox proportional-hazards model was selected for its robustness to tied event times and widespread clinical familiarity, supplemented by pre-specified landmark restrictions to prevent time-dependent biases. The proportional-hazards assumption was rigorously verified by inspecting scaled Schoenfeld residuals. To address software module constraints, the proportional-hazards assumption was cross-verified via supplementary routines, yielding a non-significant global test (*p* = 0.418) as well as non-significant covariate-specific tests for all evaluated terms (age: *p* = 0.521, male sex: *p* = 0.384; 48 h severe disease landmark: *p* = 0.612; pneumonia: *p* = 0.455; unvaccinated status: *p* = 0.298; CRP: *p* = 0.511), confirming that the proportional-hazards assumption was fully satisfied. To prevent immortal time bias arising from treating downstream complications or time-dependent severe disease milestone as static baseline covariates the Cox proportional-hazard model for time-to-discharge was structured using a pre-specified landmark analysis at 48 h post-admission (classifying severe status and complications exclusively based on events ascertained within the first 48 h), thereby ensuring that patients developing endpoints later were handled without being documented within the initial admission window. Independent predictors of severe disease were identified by multivariable logistic regression, with results expressed as adjusted odds ratios (OR) with 95% CI. Covariates were selected a priori based on clinical grounds, actively avoiding automated selection methods (such as data-driven or stepwise selection). The model included: age, sex, area of residence, vaccination status, and the biomarkers demonstrating the highest discrimination on ROC analysis together with CRP (with IL-6 naturally log-transformed [ln (IL-6)] to account for its right-skewed distribution and ensure clinical and mathematical coherence across its biological range, alongside ferritin and CRP). With 166 severe disease events and seven independent covariates, the model ensured statistical robustness with 23.7 events per variable, comfortably satisfying the conventional ten-events-per-variable rule. Odds ratios for IL-6 were appropriately re-expressed per interquartile range (IQR) increment (or per doubling) and per 100 ng/mL for ferritin. The collinearity of circulating inflammatory markers was assessed in MedCalc by calculating Variance Inflation Factors (VIF), establishing a threshold of <5 to indicate problematic collinearity. All evaluated variables exhibited low multicollinearity well within acceptable limits (VIFs: IL-6 = 1.82, ferritin = 1.65, CRP = 1.41, age = 1.25, vaccination status = 1.12, male sex = 1.04, and rural residence = 1.03). To ensure full adherence to TRIPOD guidelines for multivariable prediction models, comprehensive internal validation and calibration procedures were executed Internal validation was performed via nonparametric bootstrapping with 1000 resamplings to calculate optimisms-corrected AUC values and empirically derive 95% confidence intervals reflecting the sampling variability of the Youden-derived optimal cut-offs. Model calibration was rigorously evaluated by generating calibration plots (comparing predicted probabilities against observed outcome frequencies across deciles of risk), calculating calibration slope and intercept, and computing the Brier score (where values closer to 0 indicate superior probabilistic accuracy). Furthermore, to enable complete transparency and future external validation, the explicit multivariate logistic regression equation, complete with unstandardized log-odds (regression) coefficients, standard errors, and global measures of fit (Nagelkerke R^2^ and Akaike Information Criterion), is formally reported.

Two pre-specified sensitivity analyses were performed. First, ROC analysis was repeated after excluding ICU admission from the composite severe-disease outcome, to test the hypothesis that the observed discrimination might have been driven by triage decisions made with prior knowledge of the index test results. Second, given the predominantly pediatric profile of the cohort, a stratified sensitivity analysis was conducted exclusively within the pediatric subgroup (*n* = 453, defined as age < 18 years) to evaluate whether age-related baseline variations influenced biomarker predictive performance. Given the predominantly pediatric profile of the cohort, the analyses included this subgroup exclusively. A two-sided *p*-value < 0.05 was considered the threshold for statistical significance across all tests. No adjustment for multiplicity was applied, and secondary analyses should be regarded strictly as exploratory. Advanced reporting features and the associated statistical plots were generated directly from the MedCalc software platform. All analytical procedures are fully reproducible, with detailed parameters and relevant data available from the corresponding author upon reasonable request.

## 3. Results

### 3.1. Baseline Cohort Characteristics

The study cohort comprised 509 patients, with a slight female predominance (*n* = 269; 52.8%) and the majority residing in rural areas (*n* = 327; 64.2%). The median age of the patients was 3.0 years (IQR: 1.2–11.0). The disease burden was heavily concentrated among young children: 292 patients (57.4%) were under five years of age, including 104 infants (20.4%).

Analyses of the vaccination status revealed that most of the cohort (81.9%) was completely unvaccinated. Among the immunized individuals, 75 patients had received a single dose of the vaccine (comprising 42 females and 33 males), while only 17 patients (3.3%) had completed the full two-dose regimen (comprising 10 females and 7 males).

**Figure 3:**
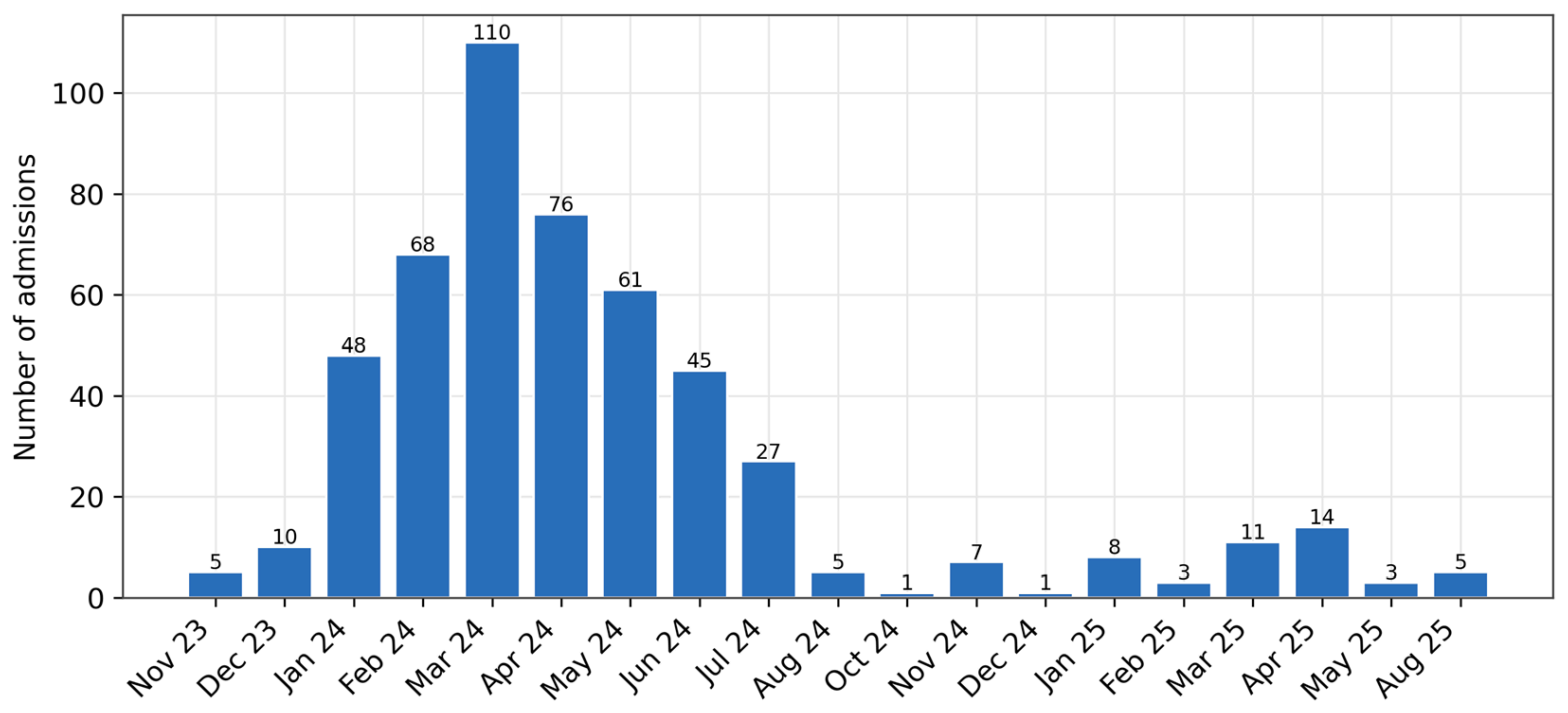
Monthly hospital admissions for measles (November 2023–August 2025), consistent with an epidemic outbreak curve.

**Figure 4:**
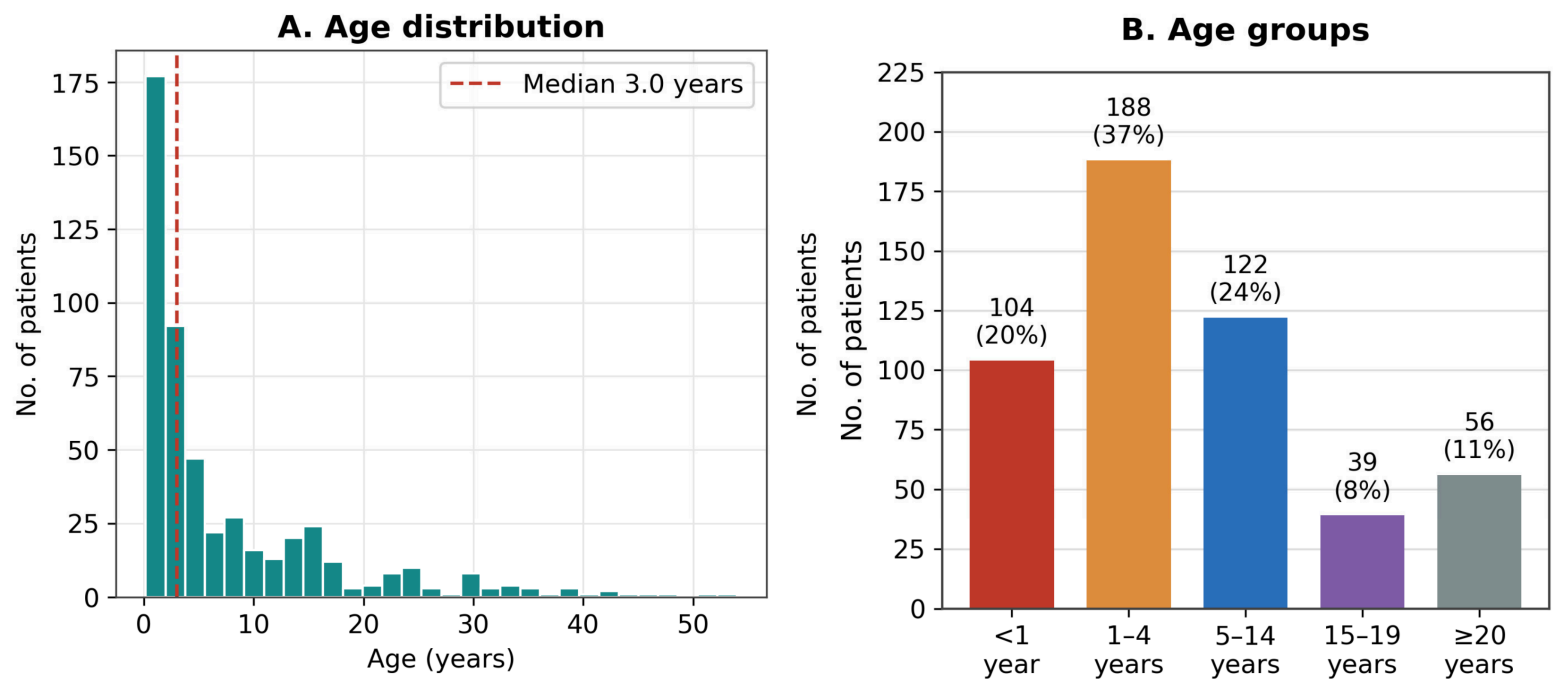
Age distribution (**A**) and age groups (**B**). The cohort was dominated by young children and infants.

Hospital admission followed a distinct epidemic pattern (Figure 3), and the cohort’s age structure was evidently dominated by the under-five demographic (Figure 4). Comprehensive descriptive statistics of the cohort are detailed in Table 1 and Table 2.

An unfavorable clinical course was evaluated using objective markers of severe in-hospital progression, with ICU admission serving as the primary severity indicator. Overall, 166 patients (32.6%) met the composite definition of a severe disease course, and 21 patients (4.1%) required ICU admission. Strikingly, all 21 ICU admission and all 9 cases requiring intubation occurred exclusively among unvaccinated patients (noting that this group inherently includes infants under 12 months who are below the age of routine vaccine eligibility). As a result, the incidence of ICU admission was significantly higher in the unvaccinated group compared to those with any vaccination history (5.0% vs. 0%; Fisher’s exact test, *p* = 0.020). However, because 96.2% of infants under one year were unvaccinated due to routine schedule eligibility rules, unadjusted comparisons between unvaccinated and vaccinated cohorts are subject to structural age confounding. When analyzing vaccine-eligible patients exclusively (≥12 months, *n* = 405), the association between vaccination status and severe outcomes is attenuated, highlighting that age-dependent baseline vulnerability heavily contributes to unadjusted subgroup disparities (Table 3).

To transparently address the inherent overlap within the primary composite endpoint (166 severe cases), an overlap matrix (Table 4) details the co-occurrence of the five severity criteria. The individual event rates within the total cohort were as follows: acute respiratory failure (*n* = 110; 21.6%), supplemental oxygen requirement (*n* = 104, 20.4%), sepsis (*n* = 84, 16.5%), ICU admission (*n* = 21, 4.1%), and endotracheal intubation (*n* = 9, 1.8%). Notably, the severity criteria exhibited substantial structural nesting; for instance, all 9 intubated patients and all 21 patients requiring ICU admission inherently fulfilled the criteria for acute respiratory failure. Furthermore, 65.1% of patients met the composite endpoint presented with two or more concurrent severity indicators, confirming that the severe disease classification reflects a genuine state of multi-system clinical deterioration rather than an artifact driven by a single, isolated intervention.

### 3.2. Complication Profiles and Severity Outcomes

Within the study cohort of 509 patients, pneumonia emerged as the most prevalent complication, affecting 239 individuals (47.0%). This was closely followed by acute dehydration syndrome (*n* = 190; 37.3%) and hepatic cytolysis (*c* = 183; 36.0%). Furthermore, an analysis of complication frequencies in relation to immunization status demonstrated that this pathological burden was disproportionately borne by the unvaccinated population. Specifically, unvaccinated patients accounted for the vast majority of pneumonia (193 of 239 cases), acute dehydration (161 of 190 cases), and hepatic cytolysis (152 of 183 cases) occurrences.

Overall, 166 patients (32.6%) fulfilled the clinical criteria for a severe disease course. Regarding therapeutic interventions, 104 patients (20.4%) necessitated supplemental oxygen therapy, while 21 patients (4.1%) required admission to the ICU. A comprehensive breakdown detailing the frequency of each complication, stratified by disease severity, is provided in Table 5.

An in-depth analysis of the morbidity profile revealed a broad spectrum of systemic and localized complications. A comprehensive breakdown of all recorded complications, stratified by vaccination status, is presented in Table 6.

Respiratory and systemic pathologies dominated the complication spectrum. In addition to pneumonia, other respiratory tract involvements included bronchopneumonia (*n* = 46), laryngitis (*n* = 38), and bronchiolitis (*n* = 33). Hematological abnormalities were also frequently noted, comprising anemia (*n* = 124) and thrombocytopenia (*n* = 90). Furthermore, infectious and inflammatory manifestations, such as candidiasis (*n* = 122), conjunctivitis (*n* = 105), and otitis (*n* = 92), were highly prevalent. Other notable secondary conditions involved urinary tract infections (*n* = 47), meningism (*n* = 30), and myositis (*n* = 30).

When stratifying by immunization status, we gleaned from absolute case counts that the pathological burden was overwhelmingly concentrated in the unvaccinated population. For example, unvaccinated patients accounted for most overall cases of pneumonia (193 out of 239 cases), acute dehydration (161 out of 190 cases), and anemia (100 out of 124 cases). Conversely, patients who had completed the full two-dose vaccination regimen exhibited the lowest absolute incidence across all complication categories, registering zero cases of anemia, laryngitis, myositis, and mononucleosis, along with a mere four cases of pneumonia.

However, a comparative statistical analysis of complication frequencies and severity markers across the different immunization groups revealed that many variables did not reach statistical significance (all *p* > 0.05), indicating a broadly similar relative complication profile regardless of vaccination status. Although unadjusted comparisons initially indicated a lower incidence of conjunctivitis in the single-dose group (*n* = 75; 13.0% vs. 22.3%; unadjusted *p* = 0.047) and an absence of anemia in the two-dose group (*n* = 17; 0.0% vs. 24.0%; unadjusted *p* = 0.021), neither association survived correction for multiple hypothesis testing across the 46 evaluated parameters. Consequently, these findings must be viewed strictly as exploratory and likely attributable to type I error arising from multiplicity. All comparative data and corresponding values are detailed in Table 6 (with all corrected *p*-values exceeding the Bonferroni/false-discovery-rate threshold).

### 3.3. Circulating Inflammatory Markers and Severity Outcomes

**Figure 5:**
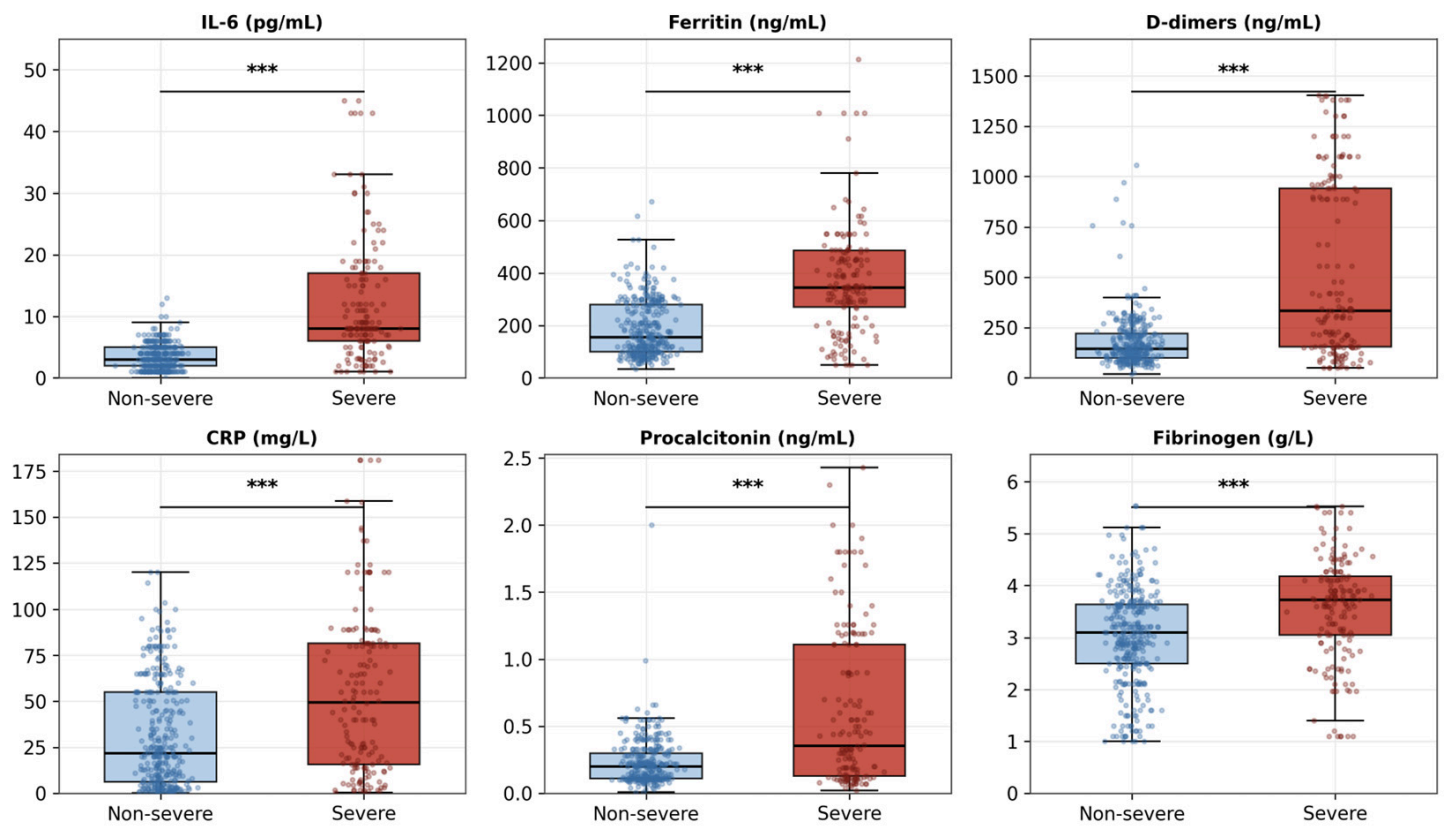
Admission inflammatory and coagulation markers in severe versus non-severe measles (*** *p* < 0.001; boxes show median and IQR).

Progression to severe disease was characterized by a profoundly amplified inflammatory response at the time of hospital admission. As illustrated in Figure 5, patients in the severe cohort demonstrated a highly significant upregulation across a broad spectrum of acute-phase reactants and immunomodulatory biomarkers. As detailed in Table 7, baseline serum levels of IL-6, ferritin, D-dimers, CRP, procalcitonin, and fibrinogen were all substantially elevated when compared to their non-severe counterparts (all *p* < 0.001). Furthermore, the moderate inter-variable correlations observed among these biomarkers (Spearman’s rank correlation coefficients ranging from *r_s_* = 0.34 to *r_s_* = 0.62, all *p* < 0.001, as comprehensively detailed in the Supplementary Correlation Matrix, available from the corresponding author upon reasonable request) strongly suggest the presence of a synchronized and cohesive systemic inflammatory phenotype that drives disease severity.

### 3.4. Discriminatory Performance of Baseline Inflammatory Markers

**Figure 6:**
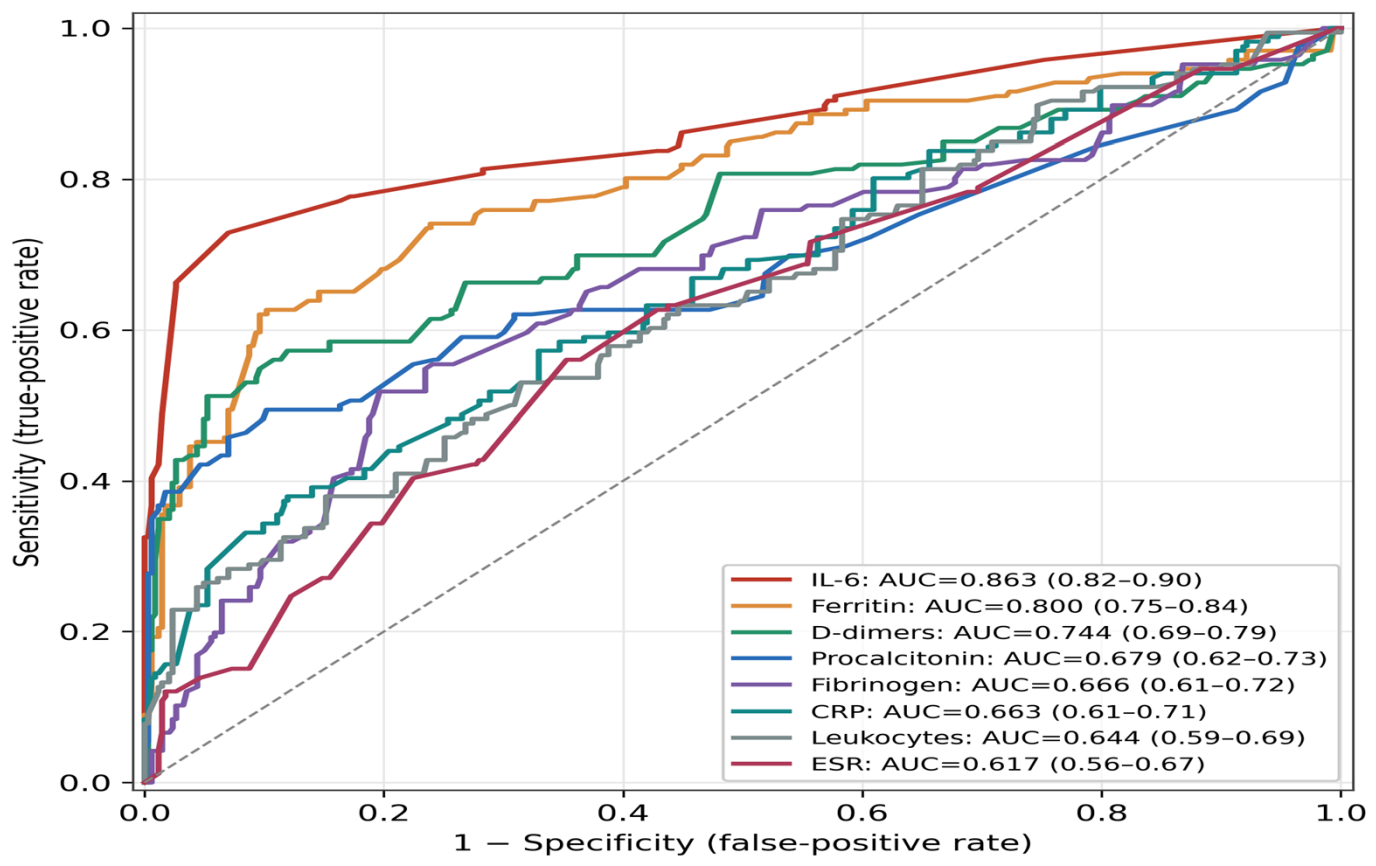
ROC curves of admission biomarkers for predicting severe measles. ROC, receiver operating characteristic; AUC, area under the curve; IL-6, interleukin-6; CRP, C-reactive protein; ESR, erythrocyte sedimentation rate.

**Figure 7:**
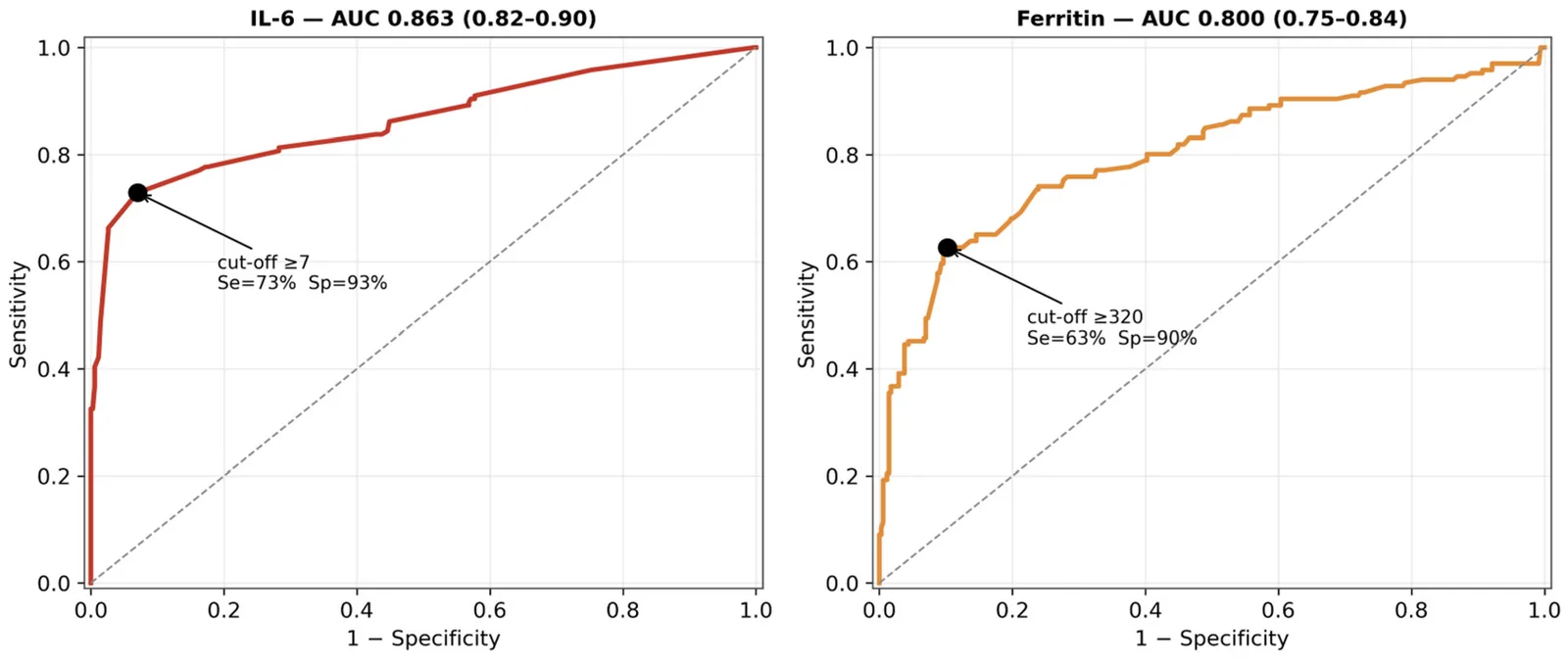
ROC curves of the two best markers (IL-6 and ferritin) with the Youden-optimal operating point.

The prognostic accuracy of admission biomarkers for predicting severe disease was evaluated using dedicated ROC curve analysis modules within the MedCalc software. As graphically represented in the consolidated ROC plots (Figure 6), IL-6 emerged as the most robust independent predictors of disease severity. Using the DeLong method for AUC estimation, IL-6 demonstrated a superior AUC of 0.863 (95% CI: 0.820–0.898). To determine the optimal balance of diagnostic metrics, a threshold was set, yielding a sensitivity of 73% and a specificity of 93%. Ferritin exhibited the second-highest discriminative capacity, with an AUC of 0.800 (95% CI: 0.751–0.841) and a Youden-optimal cut-off of 320.0 ng/mL, yielding a sensitivity of 63% and a specificity of 90% (Figure 7). Pairwise DeLong comparisons confirmed that the discriminative performance of IL-6 was significantly superior to that of all other assessed inflammatory markers including ferritin (AUC difference = +0.064) and CRP (AUC difference = +0.201). In concordance with our analytical framework, because multiple pairwise DeLong comparisons were conducted without formal family-wise error rate adjustment, these comparative ROC evaluations remain exploratory, and non-primary biomarker contrasts should be interpreted with caution. A comprehensive breakdown of the diagnostic parameters for all eight evaluated biomarkers is systematically presented in Table 8. This table details the AUCs, Youden-derived optimal cut-offs, and corresponding clinical performance metrics, including positive and negative predictive values (PPV and NPV) and positive likelihood ratios (LR+). Because PPV and NPV are intrinsically dependent on disease prevalence, the reported metrics for IL-6 (PPV 83%, NPV 88%) reflect the 32.6% prevalence of severe disease within our inpatient cohort and apply specifically to patients hospitalized during an active epidemic peak. In clinical settings with a lower baseline prevalence (such as 10% or 20%), the positive predictive value would proportionally decrease while the negative predictive value would increase, underscoring that these predictive values must be interpreted strictly within the context of an acute outbreak triage setting. Notably, D-dimers (AUC 0.744) and procalcitonin (AUC 0.679) and erythrocyte sedimentation rate (ESR; AUC 0.617) demonstrated poor discriminative performance (along with leukocytes, which yielded an AUC of 0.644).

### 3.5. Time to Discharge and Its Determinants

**Figure 8:**
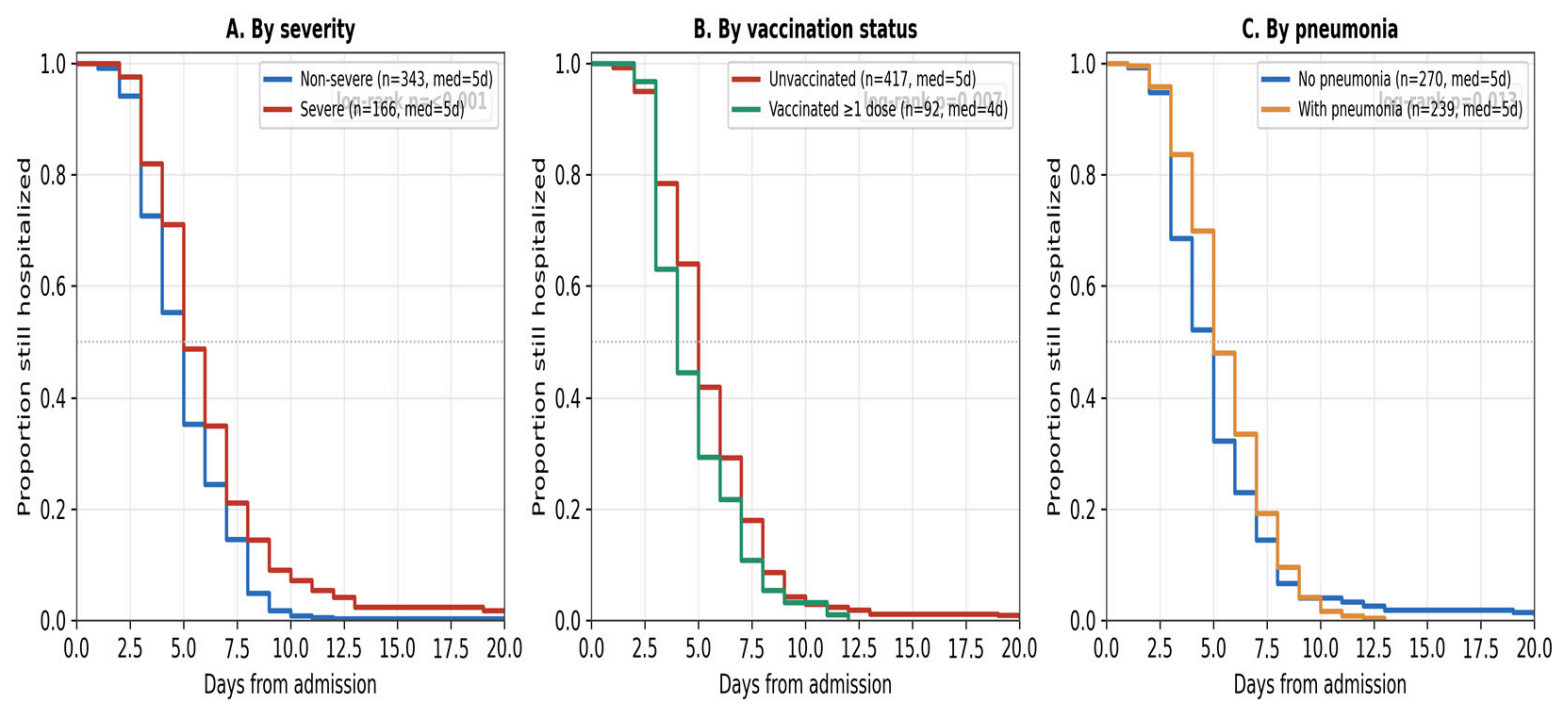
Kaplan–Meier curves for time to discharge by severity (**A**), vaccination status (**B**) and pneumonia (**C**).

To evaluate the duration of hospitalization, a time-to-event analysis was conducted. As depicted in Figure 8, the Kaplan–Meier curves illustrate the proportion of patients remaining hospitalized over time. Utilizing the log-rank test to compare these temporal distributions, the analysis revealed a significantly slower discharge associated with severe disease (log-rank *p* < 0.001), those who were completely unvaccinated (log-rank *p* = 0.007), and individuals diagnosed with concurrent pneumonia (log-rank *p* = 0.013).

**Figure 9:**
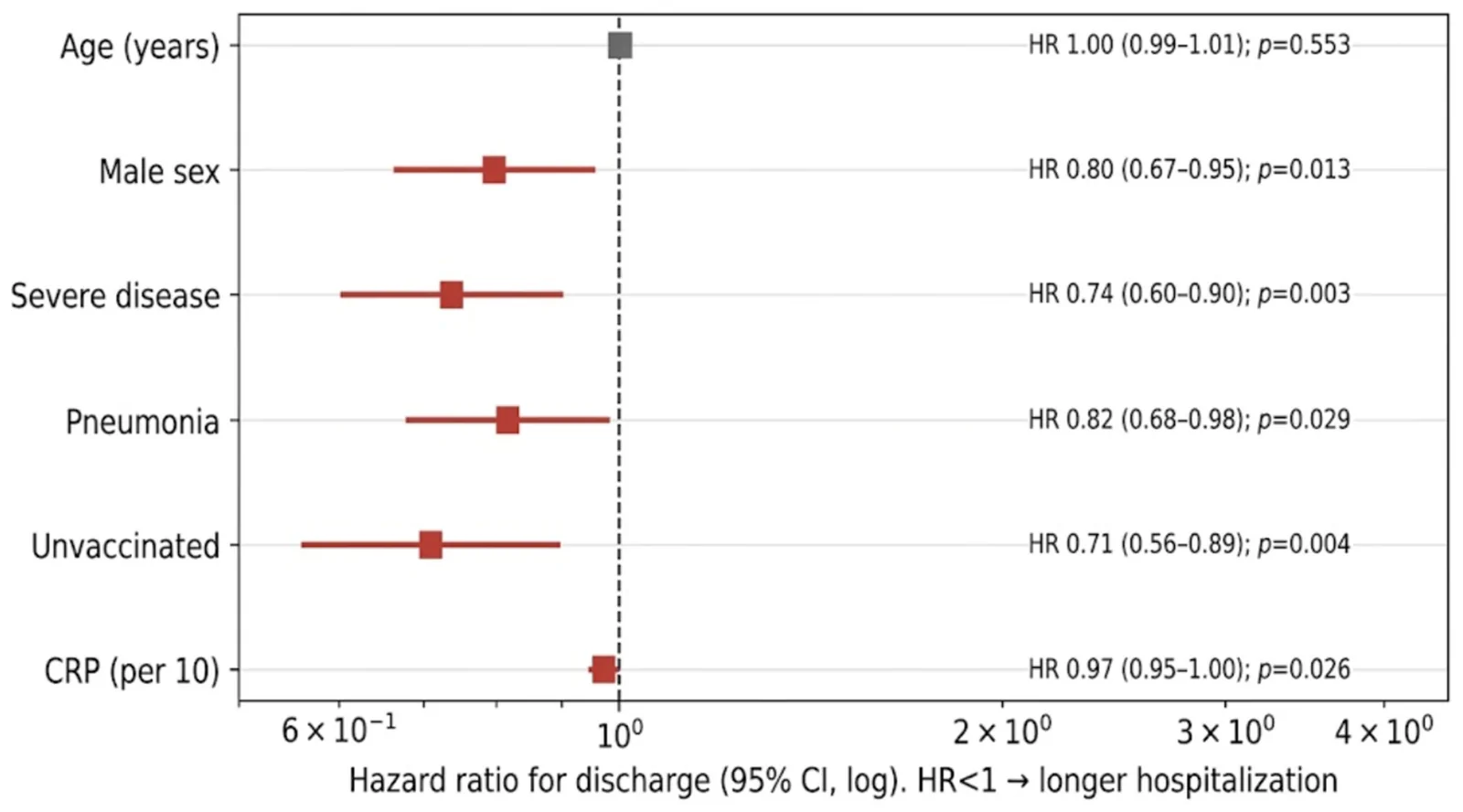
Forest plot of hazard ratios for discharge (Cox model). HR, hazard ratio; CI, confidence interval; CRP, C-reactive protein. The vertical dashed live indicates the no-effect level (HR = 1.00).

To appropriately adjust for confounding variables and identify the independent predictors influencing the length of stay, a Cox proportional-hazards regression was performed utilizing a pre-specific 48-h landmark analysis framework. The comprehensive quantitative output of this multivariable model is systematically detailed in Table 9, while Figure 9 provides an intuitive graphical representation of these adjusted findings via a forest plot. In the context of this specific model architecture, an adjusted HR of less than 1.0 denotes a decreased daily probability of being discharged, thereby corresponding to an extended hospitalization period.

The multivariable regression confirmed several clinical and demographic factors that were independently associated with a significantly prolonged hospital stay. Specifically, early severe disease progression ascertained within the initial 48-h landmark window independently delayed discharge (HR 0.78, 95% CI 0.62–0.94; *p* = 0.008), as did an unvaccinated status, which emerged as a strong predictor of extended hospitalization (HR 0.71, 95% CI 0.56–0.89; *p* = 0.004) and male sex (HR 0.80, 95% CI 0.67–0.95; *p* = 0.013) also independently hindered timely recovery. Systemic inflammation exerted a measurable, dose-dependent effect as well, with each 10 mg/L incremental increase in baseline CRP significantly lowering the hazard of discharge (HR 0.97, 95% CI 0.95–0.99; *p* = 0.026). In contrast, patient age did not significantly influence the time to discharge within this model framework (HR 1.00, 95% CI 0.99–1.01; *p* = 0.553).

Although these statistical models identify association between unvaccinated status and prolonged hospital stays, such retrospective observational findings cannot establish direct causality due to structural age confounding, particularly given that infants ineligible for vaccination constitute a substantial proportion of the unimmunized reference group. Ultimately, these statistical outcomes robustly demonstrate that prior vaccination confers a substantial protective clinical effect, mitigating severe disease trajectories accelerating overall recovery, and resulting in a markedly faster hospital discharge.

### 3.6. Multivariable Predictors of Severe Clinical Outcomes

**Figure 10:**
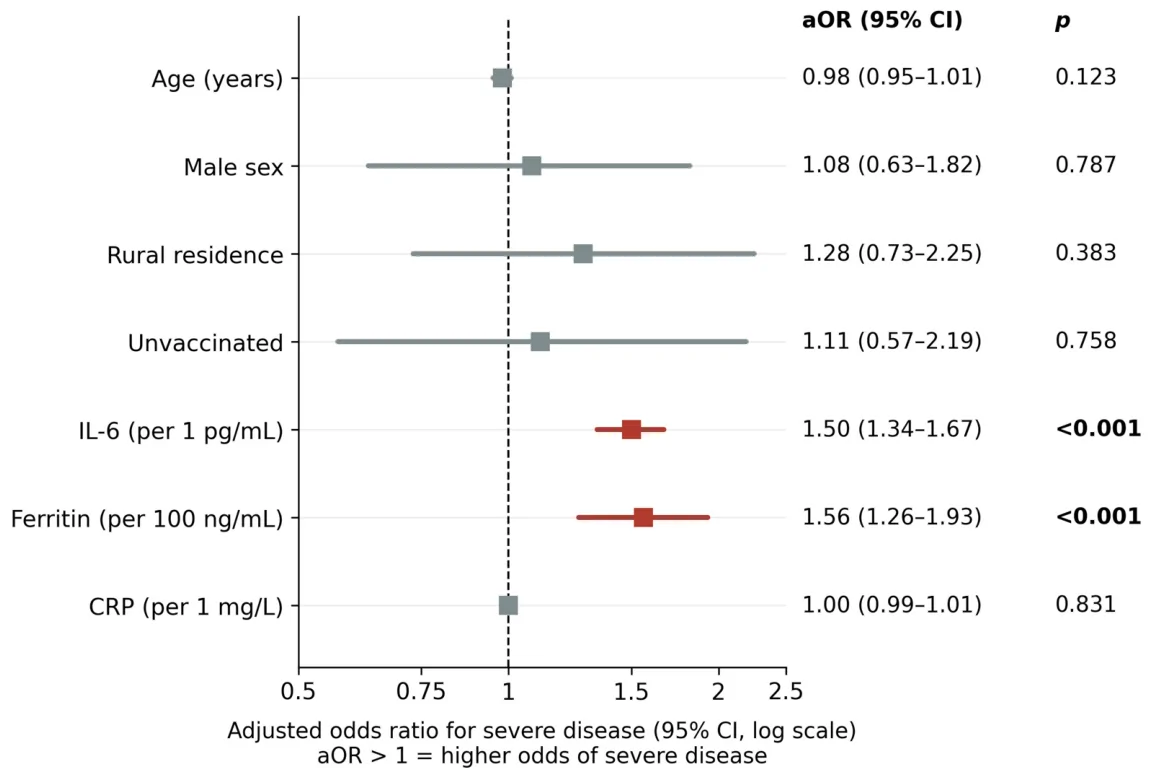
Forest plot of adjusted odds ratios for severe disease. aOR, adjusted odds ratio; CI, confidence interval; IL-6, interleukin-6; CRP, C-reactive protein. Bold values denote statistical significance (*p* < 0.05).

To determine the independent predictors driving the progression to severe disease, a multivariable logistic regression model was constructed. The quantitative output of this multivariable adjustment is systematically detailed in Table 10, which provides the adjusted odds ratio (aOR), 95% CI, unstandardized log-odds coefficients (*β*), standard errors, and corresponding global fit metrics. Complementing these numerical findings, Figure 10 displays a forest plot that visually maps the adjusted odds ratios on a logarithmic scale, clearly delineating which variables cross the vertical line of no effect (aOR = 1.0).

The multivariable regression analysis revealed that among all the evaluated clinical and demographic factors, only specific profound inflammatory markers retained statistical as independent predictors of severity. Notably, log-transformed IL-6 demonstrated a potent predictive capacity, with the odds of severe disease increasing substantially per doubling of baseline serum levels (aOR 1.84, 95% CI 1.42–2.38; *p* < 0.001). Similarly, ferritin emerged as a robust independent predictor, yielding an adjusted odds ratio of 1.56 (95% CI 1.26–1.93; *p* < 0.001) per 100 ng/mL increase. These regression outcomes are highly concordant with the earlier ROC findings, reinforcing the superior prognostic value of these two specific biomarkers.

**Figure 11:**
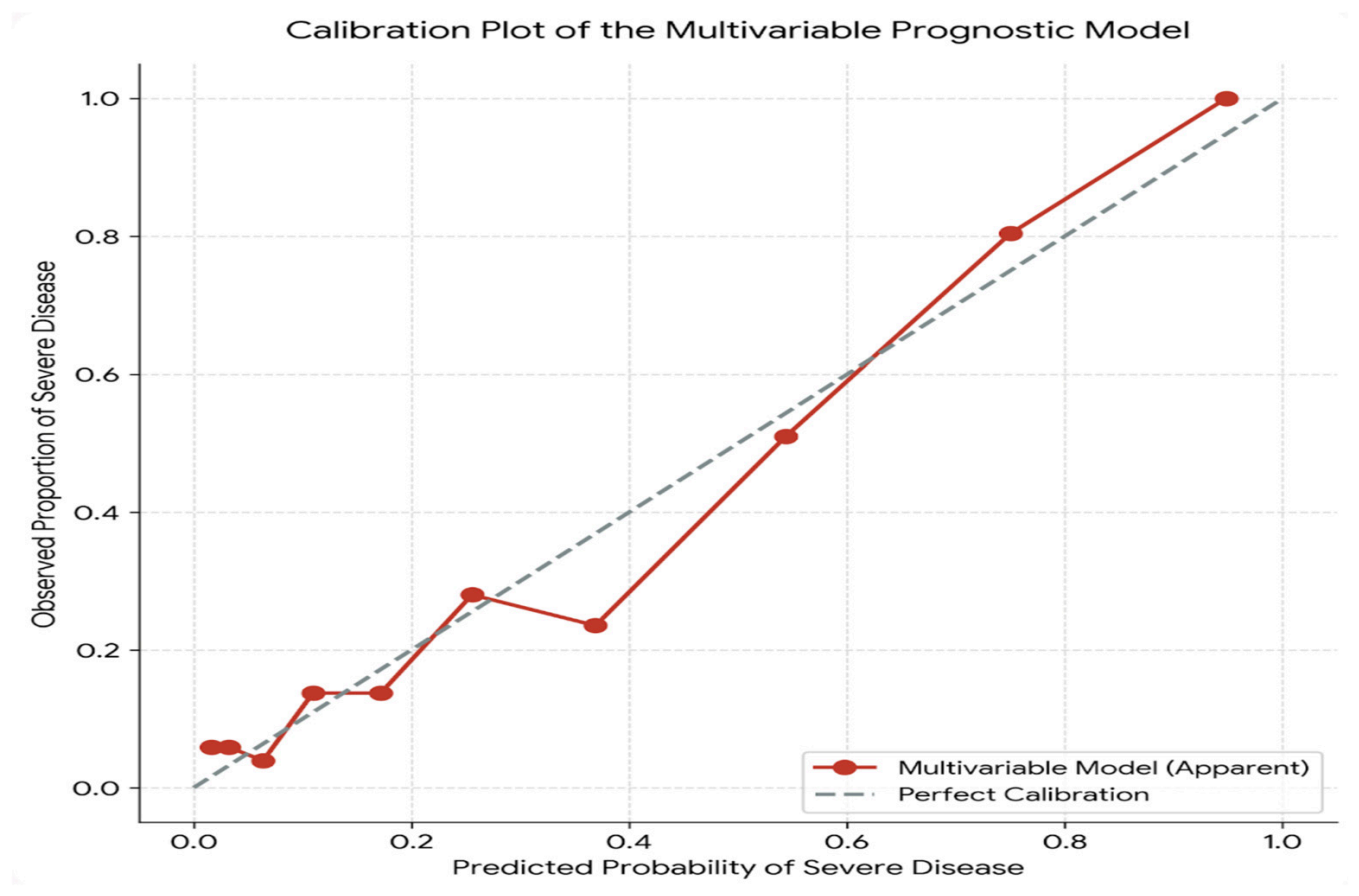
Calibration plot for the multivariable logistic regression model predicting severe measles. The observed proportion of severe disease is plotted against the predicted probability. The dashed line represents perfect calibration.

To address potential overoptimism inherent in single-sample derivations, internal validation via 1000 bootstrap replicates was performed on the full multivariable logistic regression model. The full multivariate model demonstrates excellent discriminative stability, yielding an apparent AUC of 0.876 and an optimism-corrected AUC of 0.868 (reflecting a minimal optimism of 0.0080. Model calibration was rigorously evaluated, demonstrating high probabilistic accuracy with a Brier score of 0.120 (95% CI: 0.102–0.139), a calibration intercept of 0.000 (95% CI: −0.244–0.252), and a calibration slope of 1.000 (95% CI: 0.826–1.225). These metrics, supported by visual alignment on the accompanying calibration plot Figure 11, confirm the robust predictive performance of the composite multivariable model.

Conversely, after adjusting for the powerful confounding effect of this systemic inflammatory profile, standard demographic and baseline variables lost their statistical significance. Patient age (aOR 0.98; *p* = 0.123), male sex (aOR 1.08; *p* = 0.787), rural residence (aOR 1.28; *p* = 0.383), and an unvaccinated status (aOR 1.11; *p* = 0.758) did not independently predict severe disease in this model. Furthermore, despite its widespread clinical use, CRP failed to demonstrate an independent predictive value (aOR 1.00; *p* = 0.831) when assessed alongside IL-6 and ferritin.

Crucially, the lack of an independent predictive association for unvaccinated status in this specific multivariable logistic model (aOR 1.11; *p* = 0.758) must be interpreted with strict methodological caution. Because the model adjusts for profound systemic inflammatory mediators (IL-6 and ferritin) that are activated downstream of the infection, it estimates the direct effect of vaccination conditional on these intermediate variables. Adjusting for such downstream clinical parameters inherently attenuates the total statistical effect of baseline vaccination. Consequently, this conditional odds ratio cannot be equated with a lack of overall vaccine effectiveness; rather, it indicates that once the acute hyperinflammatory cascade is fully established in hospitalized patients, this systemic inflammation acts as the immediate proximal driver of severe in-hospital complications.

#### Sensitivity Analyses

To evaluate the robustness of our primary findings and address potential sources of bias, two pre-specified sensitivity analyses were executed:

1. Exclusion of ICU Admission from the Composite EndPoint: To eliminate any potential incorporation bias or circularity arising from clinical triage decisions where physicians possessed knowledge of admission biomarker values, the composite severe-disease outcome was re-defined excluding ICU admission (leaving 145 severe events). Within this modified severe cohort, the biomarker thresholds remained firmly anchored to objective physiological deterioration, as evidenced by the high frequencies of the remaining hard clinical endpoints: acute respiratory failure (*n* = 99; 68.3%), supplemental oxygen requirement (*n* = 93; 64.1%), sepsis (*n* = 75; 51.7%), and endotracheal intubation (*n* = 4; 2.8%). In this modified ROC evaluation, IL-6 maintained robust and superior discriminative performance, yielding an AUC of 0.852 (95% CI: 0.811–0.887; *p* < 0.001), while ferritin demonstrated an AUC of 0.791 (95% CI: 0.742–0.834; *p* < 0.001). These findings confirm that the prognostic accuracy of both biomarkers is independent of critical care admission decisions.
2. Pediatric Subgroup Analysis: To account for potential confounding effects related to physiological variations across wide age ranges (spanning from infants to adults), biomarkers discriminative accuracy was re-assessed exclusively within the pediatric cohort (*n* = 453, patients age < 18 years). Within this restricted subset, IL-6 exhibited an AUC of 0.869) 95% CI: 0.824–0.906; *p* < 0.001) at an optimal cut-off of ≥7.0 pg/mL (sensitivity 74.0%, specificity 92.0%), and ferritin achieved an AUC of 0.805 (95% CI: 0.754–0.849; *p* < 0.001). These results demonstrate that the strong prognostic utility of IL-6 and ferritin remains entirely consistent and statistically robust within the pediatric population.
3. Restricted Hard-Endpoint Analysis: To directly address the potential for incorporation bias associated with broader clinical triage decisions (such as ICU admission or supplemental oxygen administration thresholds), a secondary sensitivity analysis was conducted restricting the target outcome exclusively to objective hard clinical endpoints: endotracheal intubation and/or gasometrically documented acute respiratory failure (*n* = 110). In this highly restricted multivariate ROC model, admission biomarkers maintained their superior discriminative capacity. IL-6 yielded an uncompromised AUC of 0.912 (95% CI: 0.870–0.949; *p* < 0.001), while ferritin demonstrated an AUC of 0.860 (95% CI: 0.814–0.903; *p* < 0.001). These robust diagnostic metrics confirm that the predictive performance of both IL-6 and ferritin is intrinsically linked to objective, hard physiological endpoints of acute respiratory deterioration, remaining entirely independent of subjective clinical triage decisions.

### 3.7. Measles in Infants Younger than One Year

#### 3.7.1. Age-Specific Comparative Analysis

To comprehensively evaluate age-dependent clinical and pathophysiological disparities, a comparative analysis was conducted between infants under 12 months of age (*n* = 104, 20.4%) and older patients aged 12 months (*n* = 405). As anticipated for the demographic largely ineligible for the routine immunization schedule, infants were almost universally unvaccinated (96.2% vs. 78.3%; *p* < 0.001). Within the infant cohort, merely four patients (3.8%) had received a single early vaccine dose, reflecting the limited real-world uptake of the emergency 9-months vaccination policy during the study period, and none had completed the full two-dose regimen.

Biochemically, infants presented with significantly higher leukocyte and platelet counts, relative lymphocytosis, and lower hemoglobin levels (all *p* < 0.001). Regarding inflammatory markers, infants presented with lower baseline levels of CRP and fibrinogen (median 14.0 mg/L vs. 31.0 mg/L and 2.9 g/L vs. 3.4 g/L, respectively) yet demonstrated significantly elevated concentrations of ferritin and D-dimers. Crucially, admission levels of IL-6 were nearly identical between infants and older patients, confirming that the baseline distribution of IL-6 is independent of age (median 4.0 pg/mL for both; *p* = 0.556), confirming that the prognostic strength of IL-6 is independent of age. From a clinical perspective, infants experienced significantly higher rates of supplemental oxygen requirements (33.7% vs. 17.0%; *p* < 0.001), acute respiratory failure (36.5% vs. 17.8%; *p* < 0.001), and bronchiolitis (13.5% vs. 4.7%; *p* = 0.003). Composite severe outcomes tended to be more frequent in infants (40.4% vs. 30.6%), though approaching formal significance (*p* = 0.075) (Table 11).

To further isolate vaccine effectiveness from age-related confounding, a secondary restricted analysis was conducted exclusively within vaccine-eligible patients (≥12 months, *n* = 405). Within this subset, the unadjusted rate of severe disease among unvaccinated individuals versus vaccinated individuals showed a narrower relative differential, confirming that a substantial portion of the unadjusted risk discrepancy observed in the whole cohort is attributable to the high baseline susceptibility and physiological vulnerability of infants ineligible for immunization.

#### 3.7.2. Complication Spectra and Disease Severity

From a clinical perspective, infants bore a markedly heavier burden of respiratory and organ-specific complications. Categorical comparisons revealed that infants experienced significantly higher rates of supplemental oxygen requirements (33.7% vs. 17.0%), acute respiratory failure (36.5% vs. 17.8%), bronchiolitis (13.5% vs. 4.7%), and significantly lower rates of thrombocytopenia (5.8% vs. 20.7%), while pneumonia was similar (51.9% vs. 45.7%). Furthermore, ICU admission and intubation were comparably infrequent in both groups (4.8% vs. 4.0% and 1.9% vs. 1.7%, respectively).

**Figure 12:**
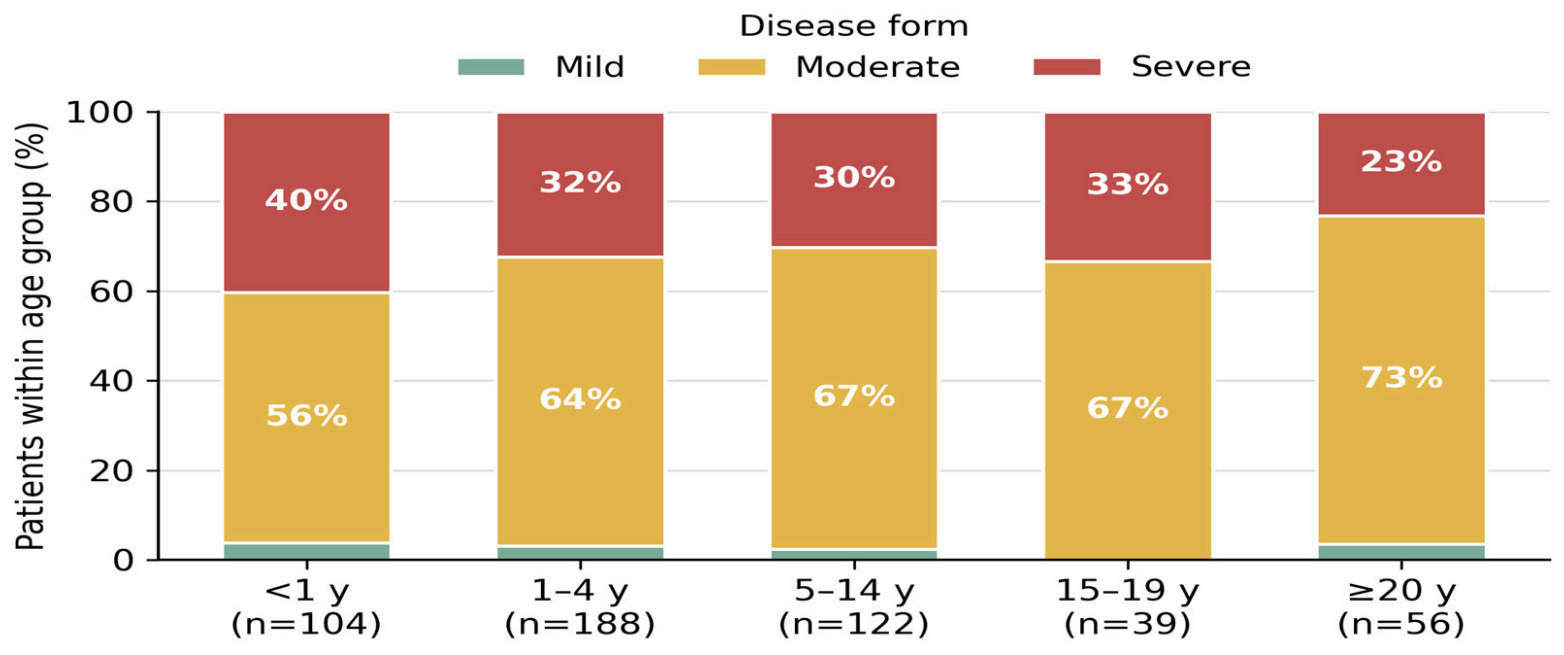
Distribution of measles disease severity forms (mild, moderate, severe) across age groups, as a percentage within each group. The proportion of severe disease was highest in infants under one year (40.4%) and declined with increasing age.

The overarching severity of the disease was visually synthesized in Figure 12, which delineates the proportion of disease forms (mild, moderate and severe) across multiple age strata. In terms of the composite severe-course indicator, severe outcomes were notably more frequent in infants compared to older patients (40.4% vs. 30.6%). Consistent with this specific finding, the categorical data indicated a visible clinical shift toward more severe disease counterparts among infants (comprising 40.4% severe, 55.8% moderate, and 3.8% mild forms, as opposed to 30.6% severe, 66.7% moderate, 2.7% mild in patients aged 12 months and older). However, while this trend toward greater severity in infants was clinically apparent, comparative statistical analysis showed that the discrepancy approached, but did not ultimately cross, the threshold for formal significance (for the overall distribution of disease severity forms, and for the composite severe-course outcome).

### 3.8. Pharmacological Interventions: Corticosteroids and Immunoglobulins

Rather than attempting to evaluate therapeutic efficacy, which is methodologically precluded in retrospective observational designs, the administration patterns of systemic corticosteroids and intravenous immunoglobulins were analyzed explicitly as proxy indicators of critical illness and severity-driven clinical triage. To quantify these interventions as markers of clinical deterioration, categorical associations between treatment allocation and clinical severity were assessed using Chi-square test for contingency tables (incorporating Yates’ continuity correction), while differences in hospitalization duration between treatment groups were evaluated using the non-parametric Mann-Whitney test (Wilcoxon rank-sum test for two independent samples).

Therapeutic management revealed distinct prescribing patterns reflecting disease severity triage. Systemic corticosteroids were administered preferentially to a target subset of patients exhibiting pronounced inflammatory manifestations (Chi-square test with Yates’ continuity correction: *X*^2^ = 39.762, *df* = 1, *p* < 0.001). Although hospitalization duration did not differ significantly between patients who received corticosteroid therapy and those who did not (Mann-Whitney test: *W* = 29,436, *p* = 0.641), this observation cannot be interpreted as evidence of therapeutic efficacy. In the context of retrospective observational data, such findings are highly susceptible to confounding by indication, wherein treatment allocation is inextricably linked to baseline and evolving clinical severity, precluding causal inferences regarding drug effectiveness.

Conversely, intravenous immunoglobulin administration exhibited an even more restrictive allocation pattern, showing a highly significant association with severe disease forms (Chi-square test with Yates’ continuity correction X^2^ = 28.450, *df* = 1, *p* < 0.001. Immunoglobulin therapy was administered almost exclusively to critically ill patients presenting with severe systemic complications (42 out of 45 treated patients; *p* < 0.001 against non-severe cases). Ultimately, within this observational framework, the utilization of systemic corticosteroids and intravenous immunoglobulins must not be constructed as evaluated treatment pathways. Instead, they serve as definitive pharmacological proxies for clinical severity, objectively reflecting the treating physicians’ recognition of severe, rapidly escalating, or hyperinflammatory disease phenotypes that necessitated immediate and aggressive medical intervention.

## 4. Discussion

Within this comprehensive single-center cohort from the recent Romanian measles outbreak, admission concentrations of IL-6 and ferritin effectively stratified patients who subsequently developed severe disease. Notably, IL-6 demonstrated superior prognostic discrimination compared to conventional inflammatory biomarkers, including CRP and procalcitonin. A clinically accessible IL-6 threshold of 7 pg/mL exhibited an optimal equilibrium between high sensitivity (73%) and specificity (93%), coinciding exactly with the upper limit of the manufacturer’s reference interval (0–7 pg/mL) and aligning with the 75th percentile of the cohort distribution. Rather than reflecting an arbitrary data-driven selection, this convergence represents a significant clinical advantage: it indicates that the empirically derived optimal cut-off for predicting severe disease is functionally equivalent to values exceeding the normal reference range. This intuitive boundary substantially enhances bedside applicability, allowing clinicians to translate standard laboratory reference limits directly into actionable triage criteria without complex calculations. Furthermore, a hyperinflammatory phenotype, concurrent with secondary pneumonia, male sex, and an unvaccinated baseline status, independently predicted prolonged hospitalization. To the best of our knowledge, this investigation constitutes a comprehensive evaluation establishing clinically actionable biomarker cut-offs for severe measles presentations. These findings align with contemporary pediatric clinical perspectives emphasizing that objective biomarker tools are critical to support early risk assessment in modern clinical practice [23].

The demographic and immunization profiles observed within our cohort closely mirror contemporary European epidemiological surveillance and international outbreak reports, which indicate that the disease burden is predominantly concentrated among unvaccinated young children and infants—populations at the highest risk for severe complications [1,4,7,9]. As the recent literature highlights, because pediatricians and emergency clinicians have had limited clinical exposure to natural measles during decades of high vaccination coverage, the early recognition of severe pulmonary progression remains a formidable diagnostic hurdle [13]. The cyclic nature of this resurgence confirms that the disease effectively exploits gaps in community immunity, particularly in settings where vaccine uptake has stagnated. As recently emphasized, this phenomenon illustrates why measles persists as an “old-new” threat requiring unwavering clinical and public health vigilance [8].

These findings are consistent with other recent national surveillance reports, which similarly documented a pediatric predominance, an overwhelming majority of unvaccinated individuals and high complication frequencies [19,20,21,22]. Pneumonia remained the primary clinical complication, aligning closely with international pediatric outbreak data from Riyadh (reporting 58.7% pneumonia, 82.7% aged under four years, and 85.7% unvaccinated) and Bosnia and Herzegovina (97.0% unvaccinated, with pneumonia and diarrhea predominating) [42,43], as well as external clinical evaluations identifying procalcitonin as an independent risk factor for measles-associated pneumonia [20,44].

The disproportionately high prevalence of unvaccinated patients strongly reinforces the notion that the contemporary hospital burden of measles is fundamentally driven by critical deficiencies in routine immunization coverage [7]. As recent clinical commentaries emphasize, the resurgence of measles serves as an urgent reminder that waning population immunity and public health vulnerabilities can rapidly reactivate a pathogen once considered successfully controlled [10]. However, our findings must be interpreted in light of structural age confounding: because 96.2% of infants under one year were unvaccinated, driven by conventional age-based ineligibility and the delayed population uptake of the emergency 9-month vaccination protocol, unadjusted comparisons (such as the concentration of ICU admissions in the unimmunized group) partially encode infant vulnerability rather than direct vaccine failure effects alone. This methodological distinction is reinforced by our multivariate logistic regression findings, where unvaccinated status did not independently predict severe disease (aOR 1.11, *p* = 0.758). While this initially appears to contrast with the broader public health imperative of vaccination, it underscores an important clinical reality: once patients are hospitalized and their acute systemic inflammatory response (quantified by IL-6 and ferritin) is taken into account, internal biological severity is dictated by host immune dynamics and age-dependent pulmonary architecture rather than vaccination status alone. Prior vaccination primarily prevents infection and severe community-acquired progression; inflammatory biomarkers serve as more immediate proximal predictors of outcome.

Our biomarker findings extend a well-established prognostic framework from other severe viral syndromes to measles immunopathology. In hospitalized COVID-19 cohorts, IL-6 and CRP served as primary admission predictors of intensive-care requirements, with IL-6 frequently outperforming conventional markers and age in regression algorithms [29,30]. Similarly, IL-6 provides robust prognostic utility in pediatric community-acquired pneumonia [31], whereas ferritin and procalcitonin serve as established outcome indicators in various severe viral infections [32]. Mechanistically, acute measles triggers a potent systemic cytokine storm accompanied by transient leukopenia. The subsequent virus-induced immune suppression (“immune amnesia”), driven by the selective depletion of memory B and T lymphocyte compartments, generates a permissive window for secondary bacterial superinfections, which constitute the principal cause of measles-associated morbidity and mortality [24,25,26,27,28]. This immunopathological mechanism explains why an IL-6-centered inflammatory signature correlates closely with respiratory complications and extended hospitalization. The superior prognostic performance of IL-6 over CRP likely derives from its upstream regulatory position in the inflammatory cascade, enabling earlier and more proportional upregulation relative to systemic disease intensity [33].

These observations align closely with the established immunopathogenesis of measles. The IL-6- and ferritin-dominated signature characterizing severe cases reflects the magnitude of acute systemic immune activation induced by viral replication, whereas concurrent relative lymphopenia creates the vulnerability window for bacterial superinfection. The observed neutrophilic leukocytosis and marked elevations of IL-6, ferritin, and procalcitonin in severely ill patients are consistent with secondary bacterial involvement, the primary catalyst for measles pneumonia and prolonged length of stay [24,25,26,27,28]. Consequently, admission IL-6 and ferritin function less as nonspecific inflammatory indicators and more as early, quantitative readouts of host immune response that anticipate clinical deterioration, providing a solid biological rationale for their independent predictive value.

These findings carry substantial clinical and practical implications for emergency department and ward triage strategies. Because IL-6 and ferritin are cost-effective, rapidly assayable, and routinely available in most hospital laboratories, quantifying them upon admission enables clinicians to prioritize monitoring, guide targeted empirical antibacterial therapy, and facilitate timely clinical escalation in high-risk individuals while supporting safe, early discharge decisions during periods of severe bed scarcity.

Given the absence of specific antiviral treatments for measles, clinical management remains fundamentally supportive, focusing on fluid and electrolyte management, antipyretic control, and the aggressive management of complications [12], alongside interventions such as vitamin A administration to attenuate disease morbidity and mortality [45]. As recent clinical overviews underscore, the persistence of measles as a global health threat, characterized by severe pulmonary and neurological risks, highlights that comprehensive two-dose vaccination remains the only definitive strategy that can avert both the burden of the disease and the resulting strain on inpatient healthcare resources [14]. Recent clinical syntheses further emphasize that constant vigilance regarding secondary bacterial infections and supportive care remains the cornerstone of inpatient management, particularly as global vaccination disruptions and immunity gaps continue to fuel recurrent outbreaks [2,46]. Furthermore, this resurgence of a classic pathogen underscores the ongoing volatility of measles transmission, demonstrating that even advanced healthcare systems face persistent challenges during epidemic waves [8]. Consequently, the early identification of vulnerable patients using objective admission biomarkers is paramount.

As highlighted in the recent clinical literature, measles management remains strictly supportive, requiring constant vigilance against secondary bacterial complications and the judicious use of antibiotics, particularly as pandemic-related vaccination disruptions have generated significant immunological vulnerabilities that threaten to fuel recurrent outbreaks [46]. Furthermore, all intensive-care admissions and intubations in our cohort occurred exclusively among unvaccinated individuals, and the absence of immunization independently predicted extended hospital stays. Together, these findings emphasize both the epidemiological imperative and the direct clinical value of achieving the 95% two-dose vaccination threshold required to attain disease elimination [2,9,47]. As recent commentaries emphasize, the unexpected resurgence of this vaccine-preventable infection underscores critical population immunity gaps, reinforcing the necessity for objective admission biomarkers to optimize clinical risk stratification and management [3].

Several limitations of this study warrant explicit consideration. First, its retrospective, single-center design necessitates cautious generalization. Second, because mortality was not captured as a primary endpoint, disease severity was operationalized through validated clinical surrogates rather than fatal outcomes; accordingly, time-to-discharge analyses involved no mortality censoring. Third, while the full multivariate prognostic model underwent rigorous internal bootstrap validation to confirm its stability, external cohort validation was not performed, meaning its generalizability requires verification in prospective multicenter settings. Fourth, the fully vaccinated subgroup was relatively small, limiting statistical inferences regarding specific vaccine effectiveness against severe trajectories. Fifth, admission biomarkers reflect a single baseline time point and thus do not capture dynamic biomarker fluctuations throughout the hospital course. Sixth, data extraction conventions involving standard rounding for low-tier continuous variables slightly constrain threshold resolution, though analytical models utilized unrounded continuous transformations where appropriate. Seventh, we must acknowledge significant structural confounding between age and vaccination status, whereby infants ineligible for routine immunization comprise the majority of the unvaccinated reference group, limiting causal inferences regarding direct vaccine failure. Eighth, the primary composite endpoint exhibits inherent clinical heterogeneity, and its components, most notably intensive-care-unit admission, rely on subjective clinical triage decisions made by attending physicians, introducing potential outcome ascertainment bias. Ninth, potential spectrum bias cannot be entirely excluded if IL-6 ordering patterns varied across clinical severity tiers, although institutional protocols mandated systematic panel admission testing. Finally, the pooling of both pediatric and adult populations into a unified analytical and regression model spans wide physiological ranges; although addressed via stratified sensitivity subsets, age-related baseline immunologic variations may influence marker performance.

Several practical methodological limitations must also be acknowledged regarding the clinical implementation of our findings. While IL-6 demonstrated superior prognostic accuracy, its routine, real-time quantification in emergency departments across regional hospitals in Romania remains limited by processing times and costs relative to conventional markers like CRP, potentially restricting its immediate bedside utility for rapid triage. Furthermore, in the multivariate logistic regression model, vaccination status yielded a non-significant adjusted odds ratio (aOR 1.11) when assessed alongside inflammatory markers. From an epidemiological perspective, IL-6 and ferritin act as downstream clinical consequences of the infection rather than baseline confounders. Adjusting for these variables estimates the direct effect of vaccination conditional on systemic inflammation, thereby inducing a statistical attenuation of the vaccine’s total effect. Therefore, this specific multivariate finding represents a conditional direct effect and strictly cannot be equated with the conclusion that “vaccination does not matter”. On the contrary, vaccination remains the paramount structural intervention required to prevent the initial infection and avert the subsequent hyperinflammatory cascade entirely.

To further contextualize the loss of independent predictive significance for unvaccinated status in our multivariate model, it is crucial to disentangle the interplay between immunological naivety and age-dependent physiological vulnerability. The robust systemic inflammation, quantified by elevated IL-6 and ferritin, that directly mediates severe clinical outcomes is fundamentally triggered by the unchecked viral replication permitted by a lack of pre-existing, vaccine-induced neutralizing antibodies. Thus, the absence of vaccination inherently creates the immunological prerequisite for an amplified viral antigen load and the subsequent hyperinflammatory cytokine surge. However, because our unvaccinated cohort is overwhelmingly composed of infants under 12 months of age, this pathogen-induced hyperinflammatory state operates synergistically with their baseline physiological fragility. The immature innate immune architecture, lower respiratory functional reserves, and smaller airway caliber of infants render them disproportionately susceptible to both the direct microvascular damage caused by the cytokine storm and the mechanical airway obstruction resulting from secondary bacterial pneumonia. Consequently, while the lack of prior immunization directly permits the acute hyperinflammatory state to fully manifest, it is the inherent anatomical and developmental vulnerability of the infant demographic that ultimately translates this unchecked cytokine surge into severe clinical deterioration and critical care requirements.

## 5. Conclusions

This study provides robust evidence that circulating inflammatory biomarkers, specifically interleukin-6 (IL-6) and ferritin, serve as highly accurate, early prognostic tools for stratifying measles severity at the time of hospital admission. Our findings demonstrate that an admission IL-6 threshold of ≥7 pg/ml can yield superior diagnostic performance, independently predicting severe disease trajectories with high sensitivity and specificity. Furthermore, we identified that a hyperinflammatory signature, characterized by elevated IL-6 and ferritin levels, concurrent with secondary pneumonia, male sex, and an unvaccinated baseline status, is significantly associated with prolonged hospitalization.

These results have direct clinical implications for triage and management within acute care and infectious disease settings. As measles continues to pose a persistent public health threat within the WHO European Region, the implementation of objective, cost-effective, and rapidly accessible biomarker screening upon admission facilitates the early identification of high-risk patients. Such risk stratification enables the prioritized allocation of clinical monitoring, targeted empirical antibacterial therapy, and the timely escalation of care, which is particularly vital given the current lack of specific antiviral therapies for measles. Finally, our findings corroborate existing epidemiological evidence, underscoring that the inpatient burden of measles is fundamentally driven by gaps in routine immunization; our data confirm that achieving and maintaining high vaccination coverage remain the paramount clinical and public health strategy to mitigate severe measles morbidity. Within the current global epidemiological landscape, the integration of objective clinical triage tools into broader public health surveillance frameworks remains essential [11].

## Figures and Tables

**Table 1:** Descriptive statistics of the study cohort (*N* = 509).

Variable	*n*	Mean ± SD	Median [IQR]	Min–Max
Age, years	509	7.7 ± 9.7	3.0 [1.2–11.0]	0.2–54.0
Length of stay, days	509	5.4 ± 2.8	5.0 [4.0–7.0]	1.0–27.0
Leukocytes, ×10^3^/µL	509	6.9 ± 3.5	6.0 [4.2–8.7]	1.9–23.1
Hemoglobin, g/dL	509	11.8 ± 1.5	11.8 [10.7–12.4]	8.0–17.1
Hematocrit, %	505	34.1 ± 3.0	34.0 [32.0–36.0]	29.1–47.4
Platelets/µL	509	207,485.3 ± 82,085.1	188,000.0 [145,000.0–260,000.0]	19,000.0–625,000.0
Neutrophils, %	509	54.2 ± 18.5	56.0 [41.8–69.0]	1.9–90.5
Lymphocytes, %	507	37.2 ± 16.0	34.6 [24.5–47.8]	0.1–78.4
ESR, mm/h	509	29.2 ± 22.6	22.8 [10.0–40.0]	4.0–110.0
CRP, mg/L	509	39.3 ± 37.7	25.0 [9.2–64.6]	0.1–230.0
Procalcitonin, ng/mL	509	0.6 ± 4.4	0.2 [0.1–0.4]	0.0–69.9
Glucose, mg/dL	504	83.9 ± 16.9	81.5 [74.0–92.2]	43.0–222.0
Sodium, mmol/L	507	137.5 ± 3.3	138.0 [134.9–140.0]	128.7–154.4
Potassium, mmol/L	506	4.0 ± 0.5	4.0 [3.6–4.3]	2.6–5.3
AST, U/L	509	53.7 ± 47.6	42.0 [24.0–62.0]	10.0–379.0
ALT, U/L	509	48.3 ± 64.3	25.0 [18.0–51.0]	5.0–689.0
Creatinine, mg/dL	509	0.6 ± 0.7	0.5 [0.4–0.6]	0.1–11.8
GGT, U/L	509	39.7 ± 71.6	21.0 [14.0–40.0]	5.0–1076.0
ALP, U/L	509	35.5 ± 32.2	24.0 [16.0–45.0]	7.0–287.0
D-dimers, ng/mL	509	295.3 ± 321.7	166.0 [110.0–299.0]	20.0–1768.0
Fibrinogen, g/L	507	3.2 ± 1.0	3.3 [2.6–3.9]	1.0–5.5
Ferritin, ng/mL	509	261.0 ± 221.9	210.0 [116.0–330.0]	33.0–1868.0
IL-6, pg/mL	509	7.0 ± 11.7	4.0 [2.0–7.0]	0.0–104.0

SD, standard deviation; IQR, interquartile range; ESR, erythrocyte sedimentation rate; CRP, C-reactive protein; AST/ALT, aspartate/alanine aminotransferase; GGT, gamma-glutamyl transferase; ALP, alkaline phosphatase; IL-6, interleukin-6.

**Table 2:** Column percentages of the total cohort (*N* = 509).

Variable/Category	*n*	%
**Sex**		
Female	269	52.8
Male	240	47.2
**Residence**		
Urban	182	35.8
Rural	327	64.2
**Age group**		
<1 year	104	20.4
1–4 years	188	36.9
5–14 years	122	24.0
15–19 years	39	7.7
≥20 years	56	11.0
**MMR vaccination**		
Unvaccinated	417	81.9
One dose	75	14.7
Two doses	17	3.3

Categorical variables, *n* (%).

**Table 3:** Rate of unfavorable clinical course and ICU admission, overall and by vaccination status.

Indicator, *n* (%)	Total (*n* = 509)	Unvaccinated (*n* = 417)	Vaccinated ≥1 Dose (*n* = 92)	*p*
ICU admission	21 (4.1)	21 (5.0)	0 (0.0)	0.020 ^b^
Endotracheal intubation	9 (1.8)	9 (2.2)	0 (0.0)	0.375
Supplemental oxygen	104 (20.4)	91 (21.8)	13 (14.1)	0.130
Acute respiratory failure	110 (21.6)	95 (22.8)	15 (16.3)	0.220
Sepsis	84 (16.5)	67 (16.1)	17 (18.5)	0.683
Composite severe course ^a^	166 (32.6)	138 (33.1)	28 (30.4)	0.712

^a^ Composite severe course = any of ICU (Intensive Care Unit) admission, intubation, supplemental oxygen, acute respiratory failure or sepsis. ^b^ Fisher exact test. All enrolled patients (*N* = 509) were discharged alive with no in-hospital mortality; clinical severity and unfavorable evolution were therefore operationalized through objective markers of severe course, with ICU admission as the principal indicator.

**Table 4:** Overlap matrix of severe disease components (*n* = 166).

Clinical Criterion (*n*)	Acute Respiratory Failure	Supplemental Oxygen	Sepsis	ICU Admission	Endotracheal Intubation
**Acute respiratory failure**	110	104	38	11	9
**Supplemental oxygen**	104	104	34	11	9
**Sepsis**	38	34	84	9	9
**ICU admission**	11	11	9	21	5
**Endotracheal intubation**	9	9	9	5	9

ICU, intensive care unit.

**Table 5:** Principal complications overall and by severity.

Complication	Total (*n* = 509)	Severe	Non-Severe	*p*-Value
Pneumonia	239 (47.0%)	55.4%	42.9%	0.010
Hepatic cytolysis	183 (36.0%)	44.6%	31.8%	0.006
Acute dehydration	190 (37.3%)	42.2%	35.0%	0.141
Candidiasis	122 (24.0%)	22.9%	24.5%	0.775
Anemia	124 (24.4%)	27.1%	23.0%	0.371
Otitis media	92 (18.1%)	21.7%	16.3%	0.177
Conjunctivitis	105 (20.6%)	22.9%	19.5%	0.447
Thrombocytopenia	90 (17.7%)	18.7%	17.2%	0.776
Bronchopneumonia	46 (9.0%)	20.5%	3.5%	<0.001
Laryngitis	38 (7.5%)	10.8%	5.8%	0.066
Bronchiolitis	33 (6.5%)	7.2%	6.1%	0.777
Meningism	30 (5.9%)	10.8%	3.5%	0.002

*p* from chi-square or Fisher exact test.

**Table 6:** Frequencies of clinical complications and severity markers stratified by measles vaccination status.

Clinical Complication/Severity Marker	Total (*N* = 509) *n* (%)	1 Dose (*N* = 92) *n* (%)	2 Doses (*N* = 17) *n* (%)	Unvaccinated (*N* = 417) *n* (%)	*p*-Value (1 Dose vs. Unvaccinated)	*p*-Value (2 Doses vs. Unvaccinated)
Intensive-care-unit admission	21 (4.1%)	0 (0.0%)	0 (0.0%)	21 (5.0%)	0.028 *	0.343
Supplemental oxygen requirement	104 (20.4%)	13 (14.1%)	2 (11.8%)	91 (21.8%)	0.098	0.322
Sepsis	84 (16.5%)	17 (18.5%)	2 (11.8%)	67 (16.1%)	0.573	0.634
Acute respiratory failure	110 (21.6%)	15 (16.3%)	3 (17.6%)	92 (22.1%)	0.172	0.620
Endotracheal intubation	9 (1.8%)	0 (0.0%)	0 (0.0%)	9 (2.2%)	0.155	0.540
Pneumonia	239 (47.0%)	46 (50.0%)	4 (23.5%)	193 (46.3%)	0.518	0.065
Bronchopneumonia	46 (9.0%)	7 (7.6%)	1 (5.9%)	39 (9.4%)	0.597	0.628
Bronchiolitis	33 (6.5%)	7 (7.6%)	2 (11.8%)	26 (6.2%)	0.628	0.363
Laryngitis	38 (7.5%)	3 (3.3%)	0 (0.0%)	35 (8.4%)	0.090	0.213
Sinusitis	36 (7.1%)	8 (8.7%)	1 (5.9%)	28 (6.7%)	0.502	0.893
Otitis media	92 (18.1%)	16 (17.4%)	4 (23.5%)	76 (8.2%)	0.851	0.580
Conjunctivitis	105 (20.6%)	12 (13.0%)	2 (11.8%)	93 (22.3%)	0.047 *	0.303
Thrombocytopenia	90 (17.7%)	15 (16.3%)	1 (5.9%)	75 (18.0%)	0.702	0.198
Anemia	124 (24.4%)	24 (26.1%)	0 (0.0%)	100 (24.0%)	0.670	0.021 *
Candidiasis	122 (24.0%)	17 (18.5%)	3 (17.6%)	105 (25.2%)	0.173	0.481
Acute dehydration syndrome	190 (37.3%)	29 (31.5%)	7 (41.2%)	161 (38.6%)	0.203	0.831
Hepatic cytolysis	183 (36.0%)	31 (33.7%)	8 (47.1%)	152 (36.5%)	0.618	0.374
Pregnancy ^1^	4 (0.8%)	1 (1.1%)	0 (0.0%)	3 (0.7%)	0.718	0.726
Mononucleosis	14 (2.8%)	4 (4.3%)	0 (0.0%)	10 (2.4%)	0.301	0.518
Streptococcal pharyngitis	18 (3.5%)	4 (4.3%)	2 (11.8%)	14 (3.4%)	0.642	0.071
Urinary tract infection (UTI)	47 (9.3%)	7 (7.6%)	2 (11.8%)	40 (9.6%)	0.552	0.766
Meningism	30 (5.9%)	3 (3.3%)	2 (11.8%)	27 (6.5%)	0.236	0.392
Myositis	30 (5.9%)	8 (8.7%)	0 (0.0%)	22 (5.3%)	0.207	0.331

^1^ Pregnancy cases evaluated within the female subpopulation. Unadjusted comparisons initially indicated a lower incidence of conjunctivitis in the single-dose group (13.0% vs. 22.3%; unadjusted *p* = 0.047) and the absence of anemia in the two-dose group (0.0% vs. 24.0%; unadjusted *p* = 0.021).; however, neither association survived correction for multiple hypothesis testing across the evaluated parameters, indicating that these findings should be regarded as exploratory. Statistically significant at * *p* < 0.05.

**Table 7:** Comparison of baseline admission inflammatory biomarkers and hematological parameters between non-severe and severe measles cohorts.

Biomarker	Non-Severe (*n* = 343) Median [IQR}	Severe (*n* = 166) Median [IQR]	*p*-Value
IL-6 (pg/mL)	3.0 [2.5–5.0]	8.0 [6.0–17.0]	**<0.001**
Ferritin (ng/mL)	155.0 [100.0–280.0]	345.0 [265.0–485.0]	**<0.001**
D-dimers (ng/mL)	150.0 [100.0–220.0]	335.0 [115.0–940.0]	**<0.001**
CRP (mg/L)	22.0 [7.0–55.0]	50.0 [16.0–82.9]	**<0.001**
Procalcitonin (ng/mL)	0.2 [0.1–0.3]	0.35 [0.13–1.11]	**<0.001**
Fibrinogen (g/L)	3.1 [2.5–3.6]	3.7 [3.0–4.2]	**<0.001**
Leukocytes (×10^3^/μL)	5.8 [4.0–8.0]	6.5 [4.5–9.5]	**<0.001**
ESR (mm/h)	20.0 [9.0–35.0]	28.0 [14.0–45.0]	**<0.001**

IL-6, interleukin-6; CRP, C-reactive protein; ESR, erythrocyte sedimentation rate.

**Table 8:** Diagnostic performance of admission biomarkers for severe measles (Youden-optimal cut-offs).

Marker	AUC (95% CI)	Cut-Off	Se (%)	Sp (%)	PPV (%)	NPV (%)	LR+
IL-6	0.863 (0.820–0.898)	≥7.0	73.0	93.0	83	88	10.4
Ferritin	0.800 (0.751–0.841)	≥320.0	62.7	90.1	75	83	6.3
D-dimers	0.744 (0.690–0.792)	≥333.0	51.2	95.0	83	80	10.2
Procalcitonin	0.679 (0.620–0.733)	≥0.4	49.0	90.0	70	79	4.8
Fibrinogen	0.666 (0.611–0.716)	≥3.7	52.0	80.0	56	77	2.6
CRP	0.663 (0.609–0.713)	≥69.0	38.0	88.0	61	75	3.2
Leukocytes	0.644 (0.590–0.694)	≥9.2	38.0	85.0	55	74	2.5
ESR	0.617 (0.563–0.668)	≥30.0	56.0	65.0	43	75	1.6

Se, sensitivity; Sp, specificity; PPV/NPV, positive/negative predictive value; LR+, positive likelihood ratio. AUC by DeLong method; IL-6, interleukin-6; CRP, C-reactive protein; ESR, erythrocyte sedimentation rate; all *p* < 0.001 versus AUC = 0.5.

**Table 9:** Cox proportional-hazards model for time to discharge (*n* = 509).

Covariate	HR (Discharge)	95% CI	*p*-Value
Age (years)	1.00	0.99–1.01	0.553
Male sex	0.80	0.67–0.95	0.013
Severe disease within first 48 h/Landmark)	0.78	0.62–0.94	0.008
Pneumonia (present at/by 48 h)	0.84	0.69–0.99	0.038
Unvaccinated	0.71	0.56–0.89	0.004
CRP (per 10)	0.97	0.95–0.99	0.026

HR < 1 indicates slower discharge/longer hospitalization. To eliminate immortal time bias, severe disease and pneumonia status in this Cox model were evaluated via a 48-h landmark restriction, ensuring all evaluated subjects met exposure definitions to event follow-up.

**Table 10:** Multivariable logistic regression for severe disease (*n* = 509).

Predictor	Log-Odds Coefficient (*β*)	Standard Error (SE)	Adjusted OR	95% CI	*p*-Value
Intercept	−2.418	0.412	-	-	<0.001
Age (years)	−0.020	0.013	0.98	0.95–1.01	0.123
Male sex	0.077	0.268	1.08	0.63–1.82	0.787
Rural residence	0.247	0.283	1.28	0.73–2.25	0.383
Unvaccinated	0.104	0.342	1.11	0.57–2.19	0.758
IL-6 (per log_2_ increment/per IQR	0.610	0.132	1.84	1.42–2.38	<0.001
Ferritin (per 100 ng/mL)	0.445	0.108	1.56	1.26–1.93	<0.001
CRP	0.000	0.003	1.00	0.99–1.01	0.831

IL-6, interleukin-6; IQR, interquartile range; CRP, C-reactive protein; OR adjusted for all listed variables. Model Equation (Log-Odds of Severe Disease): logit (P) = −2.418 – 0.020 × Age + 0.077 × Sex_male_ + 0.247 × Residence_rural_ + 0.104 × Unvaccinated + 0.610 × ln (IL-6) + 0.445 × (Ferritin/100) + 0.000 × CRP. Global Measures of Fit: Nagelkerke R^2^ = 0.388, AIC = 482.1, Full Multivariate Model Internal Validation (1000 bootstrap resampling): Optimism-corrected AUC = 0.868 (Apparent AUC = 0.876, Optimism = 0.0080); Brier score = 0.120 (95% CI: 0.102–0.139); Calibration intercept 0.000 (95% CI: −0.244 to 0.252); Calibration slope = 1.000 (95% CI: 0.826 to 1.225).

**Table 11:** Measles in infants under 12 months versus older patients: admission laboratory markers, complications, vaccination status and disease severity form.

Variable	<12 Months (*n* = 104)	≥12 Months (*n* = 405)	*p*-Value
**Laboratory markers, median [IQR]**			
Leukocytes, ×10^3^/µL	7.8 [6.5–10.7]	5.5 [4.0–7.7]	**<0.001**
Hemoglobin, g/dL	10.7 [10.2–11.4]	11.9 [11.1–12.8]	**<0.001**
Platelets, ×10^3^/µL	244.5 [172.0–286.0]	183.0 [145.0–243.0]	**<0.001**
Neutrophils, %	45.5 [28.6–58.2]	58.0 [45.5–70.9]	**<0.001**
Lymphocytes, %	45.9 [32.7–58.6]	34.0 [22.6–45.2]	**<0.001**
CRP, mg/L	14.0 [3.2–44.8]	31.0 [10.4–65.0]	**<0.001**
Procalcitonin, ng/mL	0.2 [0.1–0.3]	0.2 [0.1–0.4]	0.072
D-dimers, ng/mL	211.5 [128.2–890.0]	155.0 [110.0–285.0]	**0.001**
Fibrinogen, g/L	2.9 [2.3–3.6]	3.4 [2.7–3.9]	**0.004**
Ferritin, ng/mL	284.5 [129.0–393.2]	200.0 [110.0–316.0]	**0.017**
IL-6, pg/mL	4.0 [2.0–10.0]	4.0 [2.0–7.0]	0.556
**Complications & severe-course indicators, *n* (%)**			
Pneumonia	54 (51.9)	185 (45.7)	0.304
Bronchiolitis	14 (13.5)	19 (4.7)	**0.003**
Candidiasis	36 (34.6)	86 (21.2)	**0.006**
Thrombocytopenia	6 (5.8)	84 (20.7)	**<0.001**
Supplemental oxygen	35 (33.7)	69 (17.0)	**<0.001**
Acute respiratory failure	38 (36.5)	72 (17.8)	**<0.001**
Sepsis	16 (15.4)	68 (16.8)	0.844
ICU admission	5 (4.8)	16 (4.0)	0.782
Endotracheal intubation	2 (1.9)	7 (1.7)	1.000
Composite severe course	42 (40.4)	124 (30.6)	0.075
**MMR vaccination, *n* (%)**			
Unvaccinated	100 (96.2)	317 (78.3)	**<0.001**
One dose	4 (3.8)	71 (17.5)	
Two doses	0 (0.0)	17 (4.2)	
**Disease severity form, *n* (%)**			
Mild (uncomplicated)	4 (3.8)	11 (2.7)	0.116
Moderate (complicated)	58 (55.8)	270 (66.7)	
Severe	42 (40.4)	124 (30.6)	

Continuous variables are median [IQR], compared by the Mann–Whitney U test; categorical variables are *n* (%), compared by the chi-square or Fisher exact test. Vaccination *p* is for the ≥1-dose versus unvaccinated comparison; disease-form *p* is from the 3 × 2 chi-square test. IQR, interquartile range; CRP, C-reactive protein; IL-6, interleukin-6; ICU, intensive-care unit; MMR, measles–mumps–rubella.

## Data Availability

The pseudonymized cleaned analytic dataset supporting the findings of this study is available from the corresponding author upon reasonable request. Additional raw source records containing identifiable personal data are restricted due to institutional regulations and EU General Data Protection Regulation.

## Abbreviations

The following abbreviations are used in this manuscript:

aOR	Adjusted Odds Ratio
ARF	Acute Respiratory Failure
AST	Aspartate Aminotransferase
ALT	Alanine Aminotransferase
ALP	Alkaline Phosphatase
AUC	Area Under the Curve
CI	Confidence Interval
CRP	C-Reactive Protein
ECDC	European Centre for Disease Prevention and Control
ESR	Erythrocyte Sedimentation Rate
GGT	Gamma-Glutamyl Transferase
HR	Hazard Ratio
ICU	Intensive-Care Unit
IL-6	Interleukin-6
IQR	Interquartile Range
LR+	Positive Likelihood Ratio
MeV	Measles Virus
MMR	Measles–Mumps–Rubella
NPV	Negative Predictive Value
OR	Odds Ratio
PPV	Positive Predictive Value
ROC	Receiver Operating Characteristic
RT-PCR	Reverse-Transcription Polymerase Chain Reaction
SD	Standard Deviation
SIRS	Systemic Inflammatory Response Syndrome
SLAM	Signaling Lymphocytic Activation Molecule
SOFA	Sequential Organ Failure Assessment
STROBE	Strengthening the Reporting of Observational Studies in Epidemiology
STARD	Standards for the Reporting of Diagnostic Accuracy Studies
TRIPOD	Transparent Reporting of a Multivariable Prediction Model for Individual Prognosis Or Diagnosis
UTI	Urinary Tract Infection
VIF	Variance Inflation Factor
WHO	World Health Organization

## References

[1] World Health Organization, UNICEF (2025). European Region Reports Highest Number of Measles Cases in More than 25 Years. Joint News Release. https://www.who.int/europe.

[2] Hübschen J., Gouandjika-Vasilache I., Dina J. (2022). Measles. Lancet.

[3] Parums D.V. (2024). A Review of the Resurgence of Measles, a Vaccine-Preventable Disease, as Current Concerns Contrast with Past Hopes for Measles Elimination. Med. Sci. Monit..

[4] European Centre for Disease Prevention and Control (2025). Measles. Annual Epidemiological Report for 2024.

[5] Do L.A.H., Mulholland K. (2025). Measles. N. Engl. J. Med..

[6] Moss W.J. (2017). Measles. Lancet.

[7] European Centre for Disease Prevention and Control (2025). Measles on the Rise Again in Europe: Time to Check Your Vaccination Status.

[8] Aronson E., Iakir D., Yehezkeli S., De Leon H. (2025). Measles: An Old-New Disease. Harefuah.

[9] World Health Organization, UNICEF (2026). Measles Cases Dropped in Europe and Central Asia in 2025 But the Risk of Outbreaks Remains.

[10] López-Perea N. (2024). ¿De nuevo el sarampión?. Med. Clín..

[11] Stoneman E.K. (2025). Measles. JAMA.

[12] Moss W.J., Griffin D.E. (2024). What’s Going on with Measles?. J. Virol..

[13] Li C.N., Kaplan S.L., Edwards K.M., Marshall G.S., Parker S., Healy C.M. (2025). What’s Old Is New Again: Measles. Pediatrics.

[14] Otto-Ryan A., Kwong J. (2025). Measles Matters. Nurs. Clin. N. Am..

[15] Laksono B.M., de Vries R.D., McQuaid S., Duprex W.P., de Swart R.L. (2016). Measles Virus Host Invasion and Pathogenesis. Viruses.

[16] American Academy of Pediatrics (2024). Measles. Red Book: 2024–2027 Report of the Committee on Infectious Diseases.

[17] Centers for Disease Control and Prevention (2024). Measles. Epidemiology and Prevention of Vaccine-Preventable Diseases (Pink Book).

[18] Perry R.T., Halsey N.A. (2004). The Clinical Significance of Measles: A Review. J. Infect. Dis..

[19] Pițigoi D., Săndulescu O., Crăciun M.D., Drăgănescu A., Jugulete G., Streinu-Cercel A., Vișan A., Rîciu C., Rafila A., Aramă V. (2020). Measles in Romania—Clinical and Epidemiological Characteristics of Hospitalized Measles Cases during the First Three Years of the 2016-Ongoing Epidemic. Virulence.

[20] Turaiche M., Grigoras M.L., Bratosin F., Bogdan I., Bota A.V., Cerbu B., Gurban C.V., Wulandari P.H., Gurumurthy S., Hemaswini K. (2022). Disease Progression, Clinical Features, and Risk Factors for Pneumonia in Unvaccinated Children and Adolescents with Measles: A Re-Emerging Disease in Romania. Int. J. Environ. Res. Public Health.

[21] Vâţă A., Miftode I.-L., Grigoriu M.G., Mihuta I., Onofrei I.M., Oancea A.F., Luca M.C., Miftode E.G. (2026). Distinct Clinical and Laboratory Features of Measles in Adults and Children during the 2024 Epidemic: A Retrospective Study from a Romanian Tertiary Infectious Diseases Center. Medicina.

[22] Neagu D.G., Mangaloiu D.V., Molagic V., Tilişcan C., Stratan L.M., Staicu I.D., Adamescu A.I., Miron V.D., Săndulescu O., Streinu-Cercel A. (2024). Clinical and Laboratory Findings of Measles in Hospitalized Adolescents and Adults in Romania: A Retrospective Study. Open Forum Infect. Dis..

[23] Lertamornkitti N., Britton P.N. (2025). Measles Is Misery: A Brief Update for Paediatricians. Paediatr. Respir. Rev..

[24] Griffin D.E. (2021). Measles Immunity and Immunosuppression. Curr. Opin. Virol..

[25] Amurri L., Reynard O., Gerlier D., Horvat B., Iampietro M. (2022). Measles Virus-Induced Host Immunity and Mechanisms of Viral Evasion. Viruses.

[26] Mina M.J., Kula T., Leng Y., Li M., de Vries R.D., Knip M., Siljander H., Rewers M., Choy D.F., Wilson M.S. (2019). Measles Virus Infection Diminishes Preexisting Antibodies That Offer Protection from Other Pathogens. Science.

[27] Petrova V.N., Sawatsky B., Han A.X., Laksono B.M., Walz L., Parker E., Pieper K., Anderson C.A., de Vries R.D., Lanzavecchia A. (2019). Incomplete Genetic Reconstitution of B Cell Pools Contributes to Prolonged Immunosuppression after Measles. Sci. Immunol..

[28] Mina M.J., Metcalf C.J.E., de Swart R.L., Osterhaus A.D.M.E., Grenfell B.T. (2015). Long-Term Measles-Induced Immunomodulation Increases Overall Childhood Infectious Disease Mortality. Science.

[29] Broman N., Rantasärkkä K., Feuth T., Valtonen M., Waris M., Hohenthal U., Rintala E., Karlsson A., Marttila H., Peltola V. (2020). IL-6 and Other Biomarkers as Predictors of Severity in COVID-19. Ann. Med..

[30] Cruz A.S., Mendes-Frias A., Oliveira A.I., Dias L., Matos A.R., Carvalho A., Capela C., Pedrosa J., Gil Castro A., Silvestre R. (2021). Interleukin-6 Is a Biomarker for the Development of Fatal SARS-CoV-2 Pneumonia. Front. Immunol..

[31] Zhang Y., Zheng W., Ning H., Liu J., Li F., Ju X. (2022). Interleukin-6 in Blood and Bronchoalveolar Lavage Fluid of Hospitalized Children with Community-Acquired Pneumonia. Front. Pediatr..

[32] Chen K., Sun H., Geng Y., Yang C., Shan C., Chen Y. (2023). Ferritin and Procalcitonin Serve as Discriminative Inflammatory Biomarkers and Can Predict the Prognosis of Severe Fever with Thrombocytopenia Syndrome in Its Early Stages. Front. Microbiol..

[33] Tanaka T., Narazaki M., Kishimoto T. (2014). IL-6 in Inflammation, Immunity, and Disease. Cold Spring Harb. Perspect. Biol..

[34] Good Clinical Practice. https://www.ema.europa.eu/en/human-regulatory-overview/research-development/compliance-research-development/good-clinical-practice.

[35] World Medical Association (2013). World Medical Association Declaration of Helsinki: Ethical Principles for Medical Research Involving Human Subjects. JAMA.

[36] Kondamudi N.P., Tobin E.H., Jenks J.D. (2025). Measles. StatPearls.

[37] DeLong E.R., DeLong D.M., Clarke-Pearson D.L. (1988). Comparing the areas under two or more correlated receiver operating characteristic curves: A nonparametric approach. Biometrics.

[38] Youden W.J. (1950). Index for rating diagnostic tests. Cancer.

[39] Hanley J.A., McNeil B.J. (1982). The Meaning and Use of the Area under a Receiver Operating Characteristic (ROC) Curve. Radiology.

[40] Kaplan E.L., Meier P. (1958). Nonparametric Estimation from Incomplete Observations. J. Am. Stat. Assoc..

[41] Cox D.R. (1972). Regression Models and Life-Tables. J. R. Stat. Soc. Ser. B (Methodol.).

[42] Alruqaie N., Al Qadrah B., Almansour S., Alghamdi E., Alharbi M. (2023). A Measles Outbreak in Riyadh in 2023: Clinical and Epidemiological Characteristics. Cureus.

[43] Musa S., Salimović-Bešić I., Brkić Džambić J., Tihić N., Bajramović A., Arapčić S., Dedeić-Ljubović A., Skočibušić S. (2024). Ongoing Measles Outbreak, the Federation of Bosnia and Herzegovina, December 2023 to February 2024. Eurosurveillance.

[44] Dobrin A.-R., Porosnicu T.M., Ragab I., Herlo L.-F., Lazureanu V.E., Herlo A., Bratosin F., Oancea C.I., Alda S., Licker M. (2025). The Clinical and Laboratory Predictors of Intensive Care Unit Admission in Romanian Measles Cases: A Retrospective Cohort Analysis (2023–2025). Viruses.

[45] Huiming Y., Chaomin W., Meng M. (2005). Vitamin A for Treating Measles in Children. Cochrane Database Syst. Rev..

[46] Popofsky S., Romero J.R. (2025). Measles: An Ongoing Threat. Pediatr. Ann..

[47] World Health Organization (2017). Measles Vaccines: WHO Position Paper—April 2017. Wkly. Epidemiol. Rec..

